# Genetic variation among progeny shapes symbiosis in a basidiomycete with poplar

**DOI:** 10.1111/nph.70395

**Published:** 2025-08-07

**Authors:** Benjamin Dauphin, Maíra de Freitas Pereira, Daniel Croll, Thalita Cardoso Anastácio, Laure Fauchery, Frédéric Guinet, Maurício Dutra Costa, Francis Martin, Martina Peter, Annegret Kohler

**Affiliations:** ^1^ Swiss Federal Research Institute WSL Zuercherstrasse 111 Birmensdorf 8903 Switzerland; ^2^ Université de Lorraine, Institut national de recherche pour l'agriculture, l'alimentation et l'environnement, UMR Interactions Arbres/Microorganismes, Centre INRAE Grand Est‐Nancy 54280 Champenoux France; ^3^ Department of Microbiology/BIOAGRO Universidade Federal de Viçosa Viçosa Minas Gerais 36570‐000 Brazil; ^4^ Laboratory of Evolutionary Genetics Institute of Biology, University of Neuchâtel 2000 Neuchâtel Switzerland

**Keywords:** basidiomycete, ectomycorrhiza, genomics, mycorrhization, *Pisolithus microcarpus*, *Populus*, symbiosis, transcriptomics

## Abstract

- Forest trees rely on ectomycorrhizal (ECM) fungi for acquiring scarce resources such as water and nutrients. However, the molecular mechanisms governing ECM traits remain inadequately understood, particularly the role of intraspecific fungal variation in root‐tip colonisation and trophic interactions.
- This study examined six ECM traits using *Pisolithus microcarpus*, an ECM fungus capable of forming ECM rootlets in poplar. A collection of 40 sibling monokaryons and their parental dikaryon was analysed through genome and transcriptome sequencing to examine quantitative trait loci, gene expression and mating‐type loci.
- These findings revealed a pronounced phenotypic continuum in poplar root colonisation by sibling monokaryons, ranging from incompatible to fully compatible strains. Genetic recombination among monokaryons was demonstrated, and genomic regions potentially involved in ECM fungal traits were identified. Transcriptomic analysis revealed greater differentiation in transcriptomic profiles between fungal strains than between fungal tissues, and uncovered tissue‐specific functional responses for ECM and free‐living mycelia. Poplar exhibited distinct transcriptomic responses when interacting with different sibling monokaryons and the parental dikaryon. Allele sorting at 11 mating‐type loci confirmed the species' heterothallic tetrapolar system.
- This study advances understanding of the genetic and transcriptomic mechanisms underlying ECM symbioses, highlighting intraspecific fungal diversity's role in forest ecosystem functioning.

## Introduction

Ectomycorrhizal (ECM) fungi serve as crucial mutualistic partners within temperate and certain tropical forest ecosystems, facilitating the provision of water and nutrients to the host trees (van der Heijden *et al*., 2015; Tedersoo *et al*., 2020). Given the frequent nutrient limitations in forest soils that impede plant growth, most forest tree species depend on ECM‐fungal symbiosis for their nutritional needs. Consequently, *c*. 20 000 ECM fungi have formed mutualistic relationships with nearly 6000 plant species, encompassing both angiosperms and gymnosperms (Brundrett, 2009; Martin *et al*., 2016). The symbiotic hyphal network, referred to as the Hartig net, facilitates nutrient exchange (Genre *et al*., 2020). On the surface of plant rootlets, the dense hyphal sheath known as the mantle accumulates nutrients, whereas the extramatrical hyphal network enables the exploration and acquisition of resources within the rhizosphere. Notably, the evolutionary innovations underpinning these tree–fungi interactions have emerged independently in at least 60 transitions from saprotrophic ancestors to ECM symbionts (Martin *et al*., 2016). Most ECM fungi have lost the saprotrophic ability to decompose lignocellulose from wood and soil organic matter (Kohler *et al*., 2015). Large‐scale genome sequencing analyses have documented the loss of plant cell wall‐degrading enzymes and the emergence of new lineage‐specific symbiosis‐induced orphan genes as primary genomic features underlying these lifestyle transitions (Miyauchi *et al*., 2020). These orphan genes are potentially linked to significant proliferation of transposable elements (TEs). Although research has elucidated the functional roles of several genes involved in symbiosis (Duplessis *et al*., 2005; Hill *et al*., 2022; Wong‐Bajracharya *et al*., 2022), there is limited understanding of the role of intraspecific genetic variation in determining ECM traits, and the genomic and transcriptomic mechanisms that underpin the establishment of these symbiotic interactions at the individual level.

*Pisolithus microcarpus* (Cke & Mass.) Cunn. (Boletales, Basidiomycota) is a fungal model that is used to investigate the genetic foundations of ECM interactions. This species is geographically widespread, possesses a broad ecological niche in the Southern Hemisphere, is amenable to *in vitro* cultivation, and is capable of colonising both native and non‐native tree species, such as grey poplar (*Populus tremula × Populus alba*). Over the past two centuries, *P. microcarpus* has been extensively used in the commercial forestry industry to inoculate eucalyptus and acacia seedlings for afforestation and ornamental purposes (Cairney, 2002). Consequently, its natural geographic range has expanded from Australasia to Africa, North and South America, and Europe, in both pure and mixed tree plantations (Grand, 1976; Martin *et al*., 2002). The species exhibits tolerance to a wide range of ecological conditions, thrives in nutrient‐poor and nutrient‐rich environments (Plett *et al*., 2022; Kulmann *et al*., 2023), and is characterised by its ability to produce long‐distance exploration hyphae in the soil, facilitating access to water and nutrients in heterogeneous environments (Agerer, 2001). Its competitive capacity for host root colonisation and its ubiquitous nature render it a compelling ECM fungus for examining carbon sequestration dynamics (Plett *et al*., 2015; Hortal *et al*., 2017). A high‐quality reference genome for *P. microcarpus* has been published (Kohler *et al*., 2015), and its contiguity and annotation were subsequently enhanced in a recent comparative genomics study (Plett *et al*., 2023). This study also phylogenetically positioned the main *Pisolithus* lineages within Boletales and identified a small set of genes common to all *Pisolithus* species (13%), which are more likely to be significantly regulated during symbiosis with a host partner (Plett *et al*., 2023). The symbiotic ability of *Pisolithus* is believed to have originated from an independent saprotrophic fungal lineage and was inherited by a common ancestor of all Boletales, *c*. 50–70 million years ago (Miyauchi *et al*., 2020; Wu *et al*., 2022). Notably, the core genes involved in symbiotic interactions enable closely related *Pisolithus* species to colonise both angiosperms and gymnosperms (e.g. *P. microcarpus* and *P. tinctorius*, respectively), allowing the exploration of ancient and recent evolutionary trajectories through inter‐ and intra‐genus comparisons that have emerged for host colonisation.

**Fig. 1:**
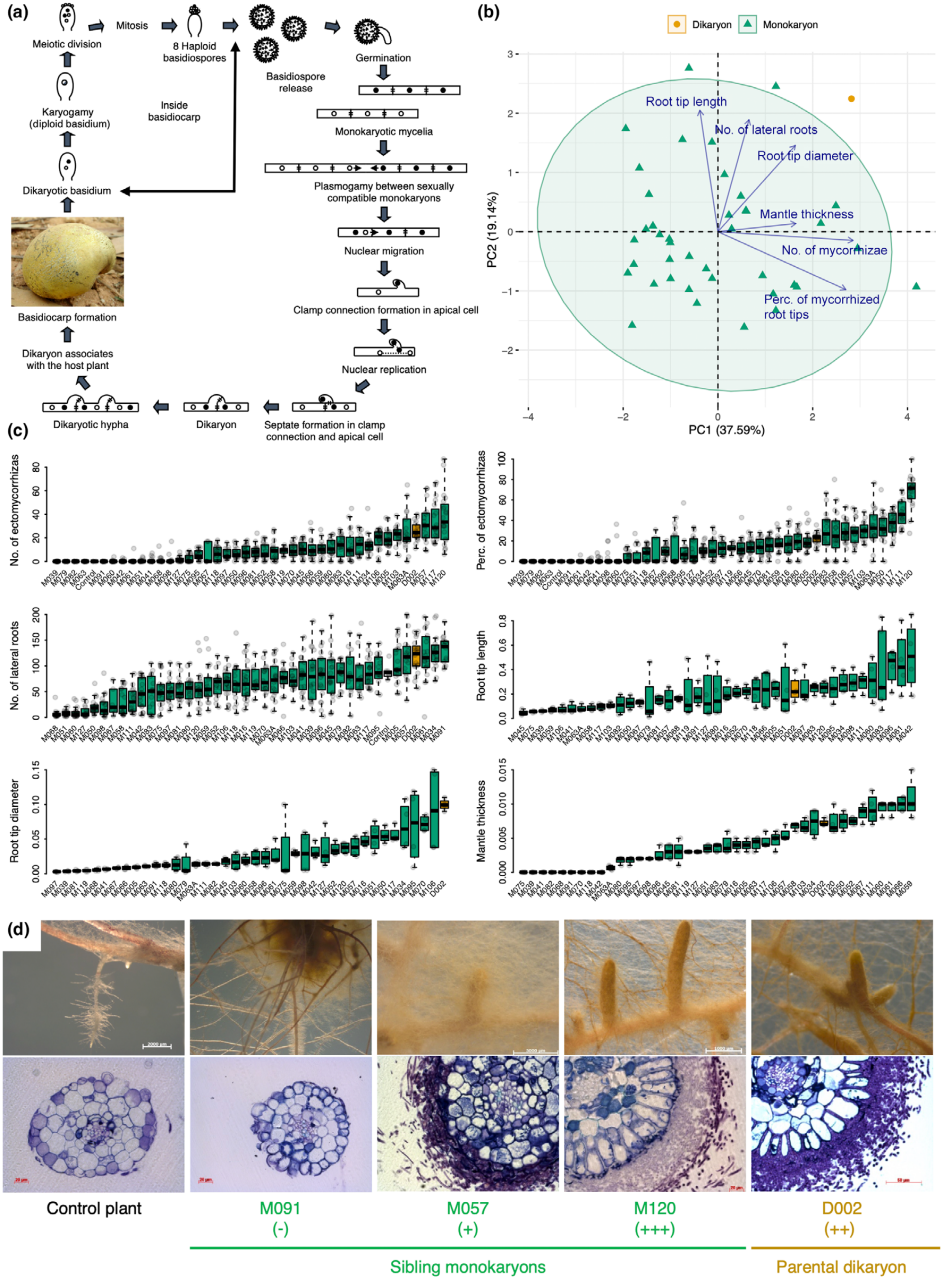
Life cycle and phenotypic variation of ectomycorrhizal (ECM) traits between the parental dikaryon and sibling monokaryon strains of *Pisolithus microcarpus*. (a) Schematic life cycle of *P. microcarpus* with monokaryotic and dikaryotic stages, as modified from (Campos & Costa, 2010). (b) Biplot of the principal component analysis (PCA) based on the six ECM traits. The parental dikaryon and sibling monokaryons are represented by orange and green triangles, respectively. The variable loadings are represented by vectors. The percentage values noted in the axis labels refer to the variance explained for each PC. The abbreviations of ECM traits are shown in Supporting Information Table S1. (c) Phenotypic variation in the six ECM traits among the parental dikaryon and sibling monokaryons. Colours indicate the karyotic stage of each strain (Table S2). (d) Colonisation and cross‐section of root tips for the parental dikaryon and sibling monokaryons selected for transcriptomic analysis. The development of the Hartig net is symbolised by minuses and pluses. Boxplots show median (center line), interquartile range (box), whiskers (1.5× IQR), and outliers (individual points). Perc., Percentage.

As for most Basidiomycota, the life cycle of *P. microcarpus* is characterised by a short monokaryotic stage and a long dikaryotic stage (Fig. 1a; Martin & Selosse, 2008; Campos & Costa, 2010). No evidence of ectomycorrhiza formation has been reported between monokaryons and tree roots under field conditions, likely due to rapid plasmogamy between sexually compatible monokaryons in the soil leading to the development of a dikaryon (Douhan *et al*., 2011). However, it was shown that isolates of *Pisolithus* monokaryons can form ectomycorrhizas with their native hosts under experimental conditions and that monokaryons exhibited considerable variability in ECM morphology and functioning, such as colour, growth, pigment production and nutrient uptake (P, Mg, Ca and K; Silva *et al*., 2007; Costa *et al*., 2010). For instance, Burgess *et al*. (1994) demonstrated that the whole spectrum of ECM formation, from fully compatible to strictly incompatible, occurred between *Pisolithus* species and *Eucalyptus grandis* based on 20 *Pisolithus* isolates. This resulted in the presence of a thick, fully developed mantle and Hartig net in the compatible *Pisolithus*–*Eucalyptus* ECM formation, while no fungal structures (mantle and Hartig net) were observed in the incompatible combinations. Moreover, intermediate levels of compatibility were observed between *Pisolithus* and *Eucalyptus* species, suggesting that ECM formation is likely to be a quantitative trait, potentially involving several gene networks in these interacting species. The genetic architecture of these ECM traits can thus be investigated using quantitative trait locus (QTL) mapping. Although this approach has been rarely applied in mycorrhizal fungi, QTL mapping has been widely used in other groups of organisms to identify chromosomal segments and associated genes that encode quantitative traits (Mackay, 2001). With advances in phenotyping methods and the unification of the ECM trait framework, it is increasingly practical to discern plant and fungal ECM traits as well as symbiotic ECM traits (Chaudhary *et al*., 2022).

Ectomycorrhizal symbiosis leads to substantial changes in plant and fungal gene expression, as shown in transcriptomic studies of host–symbiont interactions involving *P. microcarpus* (Duplessis *et al*., 2005; Plett *et al*., 2015; Wong‐Bajracharya *et al*., 2022). These changes occur at four main stages of ECM formation (Smith & Read, 2008): pre‐contact (Jambois & Lapeyrie, 2005), colonisation (Tagu *et al*., 2002), differentiation (Martin *et al*., 2001) and functioning (Pardo *et al*., 2005). Transcriptomic studies have revealed that *Pisolithus* species undergo significant upregulation of genes encoding small secreted proteins (SSPs), carbohydrate‐active enzymes (CAZymes) and effector‐like proteins during symbiosis with host tree species (Plett *et al*., 2020, 2021, 2023). These fungal genes are likely involved in host root colonisation and symbiotic signalling, as well as in the suppression of host immune responses. On the host side, *Populus* exhibits strong transcriptional reprogramming, including differential expression of genes associated with cell wall remodelling, defence suppression and nutrient exchange (Marques‐Galvez *et al*., 2024).

Investigation of the *P. microcarpus* reference genome has revealed the presence of multiple sex‐related genes, typical of a tetrapolar heterothallic mating system (Plett *et al*., 2023). In addition to the two unlinked mating‐type loci (*A* and *B* loci), six transcripts coding for pheromone receptors or pheromone activity and two mating‐related homeodomain genes have been reported in this species (Plett *et al*., 2023). Although the *A* locus is highly conserved in Basidiomycetes, the *B* locus and its flanking genes have been shown to be highly variable across closely related species with cases of gene duplication, translocation and TE insertions (Niculita‐Hirzel *et al*., 2008). Moreover, the expression levels of mating‐type genes have been shown to be heterogeneous, with some significantly upregulated in certain tissues, while others were weakly expressed throughout the life stages (de Freitas Pereira *et al*., 2017).

Phenotypic, genomic and transcriptomic data were used to examine the molecular determinants of ECM formation in *P. microcarpus* using a poplar clone as the host. Six ECM traits were assessed using time‐series data from the parental dikaryon and 40 sibling monokaryons to quantify phenotypic variation and the capacity of each strain to form ECM tissues, including the Hartig net. Subsequently, genotypic variation among the parental dikaryon and sibling monokaryons was analysed using whole genome sequencing data, and associations between phenotypic variation and newly formed allele combinations in sibling monokaryons were tested. Furthermore, transcriptomic profiles were compared between the parental dikaryon and its sibling monokaryons to determine whether they were capable of forming ECM with poplar, and gene expression analysis was conducted between the two tissue types of each selected strain, namely ECM tissue and free‐living mycelium (FLM). Additionally, sequence variability in the mating‐type loci and their flanking regions in monokaryons, relative to their parental dikaryon, was investigated. Allele segregation occurring during meiosis, along with potentially linked genomic regions involved in sexual reproduction, was also examined.

## Materials and Methods

### Tree host, parental fungal isolate and cultivation of sibling monokaryons

Grey poplar (*Populus canescens* (Aiton) Sm, *P. tremula* L. × *alba* L.; INRA clone 717‐1‐B4) seedlings were micropropagated *in vitro* and grown on half‐strength Murashige and Skoog (MS) medium (Murashige & Skoog, 1962) in glass culture tubes under a 16‐h photoperiod at 25°C in a growth chamber. To synchronise rhizogenesis, plants were first grown on MS medium containing indole‐3‐butyric acid for 1 wk, then transferred to hormone‐free MS medium for 3 wk (Felten *et al*., 2009).

A basidiocarp of *Pisolithus microcarpus* (Cke & Mass.) Cunn. (voucher specimen VIC 26495) was collected from a commercial *Eucalyptus* sp. plantation in Viçosa (Minas Gerais State, Brazil) in September 2011. The collected basidiocarp was immediately transported to the laboratory and superficially cleaned with 70% ethanol. For isolation of the dikaryon, four 5 × 5 mm fragments of the basal inner gleba of the basidiocarp were cut off and inoculated into Petri dishes with 30 ml of Modified Melin Norkrans (MMN) agar and incubated at 25°C (Marx, 1969). Pure cultures were maintained at 25°C and transferred to a new culture medium every 30 d.

Spores from the same basidiocarp were collected and stored at −70°C for a maximum of 6 months and germinated according to the method described by Costa *et al*. (2010). For spore germination, *c*. 100 mg of basidiospores was weighed and washed in 1 ml of 0.5% Tween 80 solution, centrifuged at 12 000 rpm (*c*.13 400 ***g***) for 5 min, and washed three times with distilled water. After removal of residues, 250 μl aliquots were transferred to new microtubes and underwent scarification treatment using 250 μl of 30% (v/v) sodium hypochlorite solution (20 g l^−1^ active chlorine) for 40 s. The treatment was stopped with 500 μl of 0.14 mol l^−1^ sodium thiosulphate, followed by centrifugation and two washes with distilled water. Spores were counted using a Neubauer chamber to obtain an inoculum concentration of 5 × 10^5^ spores ml^−1^.

Colonies from spore germination were transferred to MMN medium and incubated for 25 d at 28°C. Fresh mycelia were stained with SYBR Green® (diluted 1000× in 10 mmol l^−1^ KH₂PO₄, containing 18% glycerol and 1 μg ml^−1^ calcofluor white) for nuclear characterisation. Visualisation was performed using an Olympus IX50 microscope with epifluorescence (450–520 nm excitation). Colonies lacking clamp connections and containing a single nucleus per compartment were identified as monokaryotic. A collection of 40 monokaryons and the parental dikaryon was obtained and maintained on MMN medium at 28°C in permanent darkness. The isolates were also tested for *in vitro* growth on MMN medium by measuring colony radii after every 3 d of incubation.

### Ectomycorrhiza formation and phenotypic trait measurements

*In vitro* interactions were established between the hybrid poplar clone and parental dikaryon and sibling monokaryons of *P. microcarpus*. The MMN medium used had low phosphorus and nitrogen content and was lined with cellophane membranes (Brun *et al*., 1995). Ten to twelve agar discs containing fungal mycelia were placed on cellophane membranes and kept at 25°C for 20 d. Two poplar plants were transferred to the fungal mycelium. Petri dishes were incubated in a growth chamber at 25°C with 16 h of light d^−1^ for 30 d. Positive (*Eucalyptus globulus*, a native host) and negative (no fungal inoculation) controls were included in the experiment. Four replicates were performed for each isolate.

The *in vitro* system was efficient for detecting changes in poplar plant roots during contact with different *P. microcarpus* isolates. We used poplar clones with the same number of adventitious roots at the beginning, so that alterations in root morphology could be attributed to different fungal isolates. Interactions between *P. microcarpus* isolates and poplar plants could be observed within the first 7 d of contact.

ECM formation was examined by visual and microscopic observations. Every 7 d, the Petri dishes were scanned using an Epson V700 Photo Perfect device to monitor root development. The number of colonised and noncolonised lateral root tips, root length and root diameter were measured and analysed using ImageJ v.1.50 (Schneider *et al*., 2012) and SmartRoot v.4.1 (Lobet & Draye, 2013). In addition, microscopic images of ECMs produced by monokaryons and dikaryons were measured for ECM length, diameter of lateral root tips and ECMs, mantle thickness and epidermal cell length and width. The presence, absence and developmental stages of the Hartig nets were also assessed (Burgess *et al*., 1994).

ECM rootlets were dehydrated using consecutive treatments with ethanol (10, 30 and 50%) for 1 h and fixed in 70% ethanol at 4°C for 24 h. The samples were incubated in 80% and 95% ethanol solutions and in absolute ethanol for 1 h before infiltration. Infiltration was performed using Technovit 7100 resin (Kulzer and Co. GmbH, D‐6393 Wehrheim, Germany), using an increasing mixture of ethanol and resin with hardener I (benzyl peroxide) for 2 h. The samples were transferred to moulds with 100% inclusion solution (Technovit 7100 resin with hardener II) for 2–4 h. Polymerisation was performed at room temperature. Cross sections (5 μm) were cut using a rotary microtome (Epredia HM 355S; Thermo Fisher Scientific, Waltham, MA, USA) and stained with 0.05% toluidine blue. The cross‐sections were then examined using an inverted microscope (Axio Observer Z1; Zeiss) equipped with a digital camera. Photographs were taken and analysed using AxioVision v.4.8.2 software. The karyotic stage of each strain was assessed under microscopy using nuclei staining. Three to four independent replicates were performed for each ECM morphology measurement. In total, we assessed and analysed six ECM‐related traits (Supporting Information Table S1).

### DNA extraction, library preparation and genome resequencing

Genomic DNA of the parental dikaryon and its sibling monokaryons was extracted using the Wizard Genomic DNA Purification kit (Promega) and purified using the CHROMA SPIN columns of the Clontech kit (Promega) according to the manufacturer's instructions. DNA was quantified by spectrophotometry (Qubit; Thermo Fisher Scientific, Waltham, MA, USA), and the quality was assessed on agarose gel (1.5%). A minimum of 0.5 μg of DNA was sent to the INRA GeT‐PlaGe sequencing facility (Toulouse, France) for library preparation using the Illumina TruSeq Nano Kit. Sequencing of the flow cell was performed on an Illumina HiSeq 2500 sequencer using the HiSeq TruSeq SBS sequencing kit v.4, following a 2 × 125 bp indexed run recipe at the INRA GeT‐PlaGe sequencing facility (Toulouse, France).

### Read mapping, variant calling, and polymorphism and genotype filtering

We inspected read quality using FastQC v.0.11.4 (Andrews, 2010) and performed quality trimming of raw reads using fastp v.0.20.0 (Chen *et al*., 2018) with a minimum length of 80 base pairs (bp). Trimmed reads were mapped onto primary scaffolds of the *P. microcarpus* reference genome v.2 (https://mycocosm.jgi.doe.gov/Pismi2/Pismi2.home.html) of strain 441 (Plett *et al*., 2023) using Bwa‐Mem v.0.7.17 with default parameters (Li, 2011). The resulting BAM files were sorted by position, indexed and filtered to a minimum read mapping quality of 20 using Sambamba v.0.8.0 (Tarasov *et al*., 2015). Duplicates were removed using Picard v.2.23.8 (Broad Institute, 2021).

We used Genome Analysis Toolkit (Gatk) v.4.1.2 and HaplotypeCaller for variant discovery (McKenna *et al*., 2010). We analysed variants in the combined monokaryon and dikaryon datasets with ploidy set to two. We did this to simultaneously call the parental dikaryon and its sibling monokaryons and to identify and remove putative paralogous regions in the monokaryons. Individual SNP calls were merged and jointly genotyped using the Gatk GenotypeGVCF tool. We retained only bi‐allelic single nucleotide polymorphisms (SNPs) and filtered the joint Variant Call Format (VCF) file using hard filtering parameters according to GATK best practice recommendations (van der Auwera *et al*., 2013). The following thresholds were applied for SNP filtering: AN ≥ 50, QUAL ≥ 5000, FS ≥ 60.0, SOR ≥ 3.0, QD ≥ 20, MQ ≥ 50.0, ReadPosRankSum, MQRankSum and BaseQRankSum each comprised between −2.0 and 2.0. Next, low‐quality genotype calls were removed using BCFtools v.1.9 (Li, 2011) based on a genotype quality score of 20 and a minimum sequencing depth of eight reads (Carson *et al*., 2014). More stringent filtering was applied to the VCF file for missing data (SNPs with ≥ 10% missing calls were excluded) and minor allele frequency (MAF ≥ 0.10) using VCFtools v.0.1.16 (Danecek *et al*., 2011) to exclude poorly supported SNPs in downstream analyses. We then reduced the SNP set by randomly selecting a single SNP per 1 kilobase pair (kbp) of the reference genome sequence using VCFtools.

### Genetic map construction, linkage groups and quantitative trait locus analysis

We used genome‐wide SNP data at a 1‐kbp distance containing the parental dikaryon and sibling monokaryons and performed an additional quality assessment of genotypes before genetic map analysis (Croll *et al*., 2015). We excluded SNPs that were homozygous or had a missing call in the parental dikaryon and removed those that were heterozygous in a single monokaryon. We then inspected the quality of the monokaryon genotypes for problematic double‐crossings at very closely spaced markers. To this end, we searched for consistently switched alleles across monokaryons and recorded suspicious genotypes when alleles were consistently inverted; that is, nearly all monokaryons (> 30) had a double crossing over at the same position, which is unlikely. We further checked putative switched alleles using the error logarithm of the odds (LOD) scores (Lincoln & Lander, 1992), as implemented in the R package qtl v.1.70 (Arends *et al*., 2010), and found no evidence for erroneous genotypes in the filtered SNP set based on pairwise linkage. To be conservative with possible random genotyping errors, we subsequently required that any double crossover in a monokaryon be spanned by a minimum of five consecutive SNPs. Following these additional filtering steps, we retained 13 140 SNPs across monokaryons for downstream analyses.

To investigate genotypic variation and potential groupings among strains, we performed a principal component analysis (PCA) on monokaryons based on the 1 kb‐filtered SNP set using the R package SNPRelate v.1.36.0 (Zheng *et al*., 2012). Missing genotypes were imputed using the dosage mean as implemented in the snpgdsPCA function (Patterson *et al*., 2006). The first three PCs, summarising the highest explained variance along factorial axes, were inspected and retained in the data analysis. To gain insight into the overall recombination among monokaryons, we generated a Neighbor‐Net network with uncorrelated *p* distances using SplitsTree v.4.19.2 (Huson & Bryant, 2006). We visualised the network with equal‐angle splits and performed a recombination test within the monokaryons using the Φ test implemented in SplitsTree (Bruen *et al*., 2006). The test identified evidence of recombination based on the genetic similarities of neighbouring SNPs in the matrix. We ran the test with the default settings of a 100‐character window size on the SNP set.

We estimated the recombination fractions among all pairs of retained SNPs using the est.rf function of the R package qtl. We defined the maximum number of iterations as 10 000 and the tolerance threshold as 1 × 10^−6^. We used the same settings to assess the genetic distance in centimorgans (cM) between each SNP pair on each scaffold using the est.map function of the R package qtl. We then checked for genotyping errors using the calc.errorlod and top.errorlod functions in the R package qtl and found that no marker exceeded the LOD score cut‐off of 4. We visualised the proportion of missing genotypes using the entropy method, as implemented in the plotInfo function of the R package qtl. We inferred linkage groups based on a maximum recombination fraction of 0.35 and minimum LOD scores of 6, 8 and 10. Finally, we visualised the quality assessment data as well as the gridded genotype data, genetic map and linkage groups using functions supported in the R package qtl.

Quantitative trait locus analysis was performed to investigate the genetic basis of ECM traits using the R package qtl. We computed conditional genotype probabilities with a maximum distance of 0, 1 and 5 cM using the calc.genoprob function, and applied simple interval mapping with the normal model and EM algorithm for each ECM trait. We defined significance thresholds using permutation tests (*n* = 1000 permutations) and applied an exploratory threshold of α = 0.40 for identifying putative QTL regions and carried out QTL analysis on the mean values of technical replicates for each ECM trait. We inspected the normality of each ECM trait using a Shapiro–Wilk normality test and applied a transformation, when appropriate, using a Box–Cox power transformation.

### Detection of copy‐number variations and association with ectomycorrhizal traits

We searched for segmental deletions and duplications in the monokaryons against the reference genome 441 v.2 using CNVcaller (Wang *et al*., 2017). CNVcaller uses the normalised read depth of read alignments to identify high‐confidence copy number variation (CNV) regions. The window size for CNV calling was set to 1000 bp, according to the recommendations based on sequencing coverage. The lower and upper GC content limits were set to 0.2 and 0.7, respectively, and the upper limit of the gap content was set to 0.5. We required a minimum Pearson's correlation coefficient between two adjacent nonoverlapping windows to be > 0.4, as recommended for a sample size between 30 and 50 strains. We also specified the minimum frequency (‐f 0.1) of gain/loss individuals and the number of homozygous (‐h 3) gain/loss individuals to define a candidate CNV window as recommended. CNV regions were further filtered based on the normalised read depth. Deletions and duplications were retained if the normalised read depth was < 0.2 and > 1.8, respectively. CNV regions called for reference genome 441 were aligned against the reference genome for validation, which showed possible issues in read alignment and/or local assembly quality. Therefore, gene deletions and duplications among monokaryons were retained only if the reference strain showed a normalised read depth between 0.6–1.4 for the same region. A gene was considered to be affected by duplication or deletion if > 50% of the coding sequence length overlapped with a CNV region. One CNV region may contain several duplicated or deleted genes. We filtered out if the minor allele frequency was less than 0.15 to reduce false positives, based on the assumption that the probability of having false positives at the same genomic locus from multiple samples is very low. In other words, we analysed the CNVs only if CNV were observed in at least six individuals out of 40 (i.e. allele frequency equal to 6/40 = 0.15). The results were visualised using the R package ggplot2 v.3.3.0 (Wickham, 2016).

Next, we fitted generalised additive models (GAMs) to investigate the relationship between the CNV regions and ECM traits. For each CNV region, we assigned the locus position using the middle physical distance ((stop.pos – start.pos) + start.pos) and modelled the phenotypic variation as a function of the categorical levels of CNV, that is treating CNV as a factor with three possible states: Deletion, Normal or Duplication. We assessed the significance of the GAM by comparing the full model with the null model using the ANOVA‐*F* test. The significance threshold was set at *P* < 0.05. In addition, we computed the model's predicted values along with 95% confidence intervals (CIs) to quantify the uncertainty around the predictions. We summarise and visualise the results using box plots and model predictions.

### RNA extraction, library preparation and transcriptome sequencing

Total RNAs were extracted from ECM tissues and FLM of the parental dikaryon (D002) and three selected sibling monokaryons (M057, M091 and M120) using a CTAB‐ and lithium chloride (LiCl)‐based method (Chang *et al*., 1993). We selected these monokaryon strains based on their ability to form ECMs with poplar clones. Total RNA was quantified and integrity was checked using an Experion Automated Electrophoresis Station (Bio‐Rad, Hercules, CA, USA). The preparation of 24 libraries and sequencing of 2 × 125 bp Illumina HiSeq 2500 mRNA (RNA‐Seq) in three lanes was performed at the INRA GeT‐PlaGe sequencing facility (Toulouse, France). Three replicates were performed for each fungal strain.

### Transcript mapping and gene expression analysis

We aligned quality‐trimmed raw reads to either *P. microcarpus* 441 v.2 primary scaffolds or *P. trichocarpa* v.4.1 reference transcripts (https://phytozome‐next.jgi.doe.gov/) using CLC Genomics Workbench v.21.0.3 (Qiagen, Hilden, Germany). The following CLC genomic workbench parameters were used for read mapping: minimum length fraction 0.9, minimum similarity fraction 0.8, mismatch cost = 2, insertion cost = 3, deletion cost = 3, and the maximum number of hits for a read was set to 10. We determined the number of unique reads and total number of mapped reads for each transcript, along with the normalised transcripts per million (TPM). We used the number of unique reads to calculate differentially expressed genes (DEGs) between conditions (i.e. strains and tissues) using the R package deseq2 v.1.42.0 (Love *et al*., 2014). We applied a regularised log transformation of the count data using the rlogtransformation function and carried out PCA on the transformed data using the plotPCA function. We computed a distance matrix on the transformed count data to visualise pairwise dissimilarities between samples and replicates.

Genes with statistically significant differences in expression were selected based on a false discovery rate (Benjamini & Hochberg, 1995) of 0.05 and a fold change of log_2_ > 1. We visualised significantly differentially upregulated and downregulated genes using Venn diagrams generated with the online tool available at http://bioinformatics.psb.ugent.be/webtools/Venn/. We performed a hierarchical clustering analysis on significantly differentially upregulated and downregulated genes and generated heatmaps using the Morpheus online tool at https://software.broadinstitute.org/morpheus/. We carried out functional annotation of the DEGs according to the JGI annotations (mycocosm.jgi.doe.gov), including predicted gene ontology (GO), EuKaryotic Orthologous Groups (Tatusov *et al*., 2003), protein family clarification using the InterPro database (Blum *et al*., 2021), Carbohydrate‐Active enZYme (Cazy; Cantarel *et al*., 2009) and secondary metabolism genes and cluster prediction (Khaldi *et al*., 2010). We summarised the top candidate DEGs in ECM tissues of *Pisolithus microcarpus* strains D002, M057 and M120 compared to their respective FLM tissues, including SSPs, as they represent an important class of fungal effector proteins known to play crucial roles in symbiotic interactions (Kohler *et al*., 2015; Martin *et al*., 2016; Plett *et al*., 2020, 2023).

### Gene variant analysis of the mating type loci

To better understand the mating system of *P. microcarpus*, we investigated gene variant distribution of the mating type loci based on our *de novo* genome assemblies of monokaryon strains. We used Spades v.3.14.1 to assemble genomic scaffolds based on paired‐end Illumina sequencing data (Bankevich *et al*., 2012). SPAdes relies on the read error correction module and builds contigs in a stepwise manner by increasing the kmer lengths. We set a kmer range of 21, 33, 45, 67 and 89 and specified the careful option to reduce mismatches and indel errors in the assembly. We used Quast v.5.1.0 to compute genome assembly statistics for each strain (Gurevich *et al*., 2013). We searched for mating type‐related genes using the reference strain 441 v.2 available on the MycoCosm portal (https://mycocosm.jgi.doe.gov) and performed a blastn to identify *de novo* assembled scaffolds that spanned the mating type loci of the *P. microcarpus* reference genome strain 441 (Camacho *et al*., 2009). Once matching scaffolds were identified in individual *de novo* genome assemblies, the sequences of each locus were aligned using Mafft v.7 with default parameters (Katoh *et al*., 2019). We computed pairwise sequence difference matrices using the R package Biostrings v.2.70.3, and visualised pairwise synteny blocks using the R package Decipher v.2.30.0.

## Results

### Large phenotypic variation among progeny monokaryons and dikaryons

We conducted a mycorrhization experiment using *P. microcarpus* and a hybrid poplar (*Populus tremula* × *alba*, INRA clone 717‐1‐B4) and found substantial phenotypic variation in six ectomycorrhiza‐related traits (ECM traits) among sibling monokaryons compared with their parental dikaryon (Table S1). Although the homokaryotic stage is short (Fig. 1a), we successfully maintained strains of *P. microcarpus* at the monokaryon stage in pure culture after spore germination (Fig. S1). The ability to form ectomycorrhizas was highly variable between strains, ranging from 0 to 68.0 for the mean percentage of mycorrhized root tips, 0–37 for the mean number of mycorrhizae, and 0–107 μm for the mean mantle thickness (Fig. 1b,c; Table S2). The other ECM traits also showed high variability among strains, ranging from 6.7 to 131.6 for the mean number of lateral roots, from 0.46 to 5.13 mm for the mean root tip length, and from 27.5 to 997.5 μm for the mean root tip diameter (Fig. 1c; Table S2). ECM traits were not highly correlated with each other (Figs 1b, S2), nor with colony growth rate, based on pairwise Pearson correlation coefficients. Our PCA of the six ECM traits revealed dissimilarities between the parental dikaryon and sibling monokaryons, with 56.7% of the total phenotypic variance explained by the first two PCs (Figs 1b, S3). The number and percentage of ECMs were the two traits that contributed the most to phenotypic variation in the PCA (Fig. S3).

Interestingly, we observed a phenotypic continuum for each ECM trait with no distinct groupings among the monokaryons (Fig. 1c). Additionally, the parental dikaryon exhibited phenotypic variation within the range observed in the monokaryons. For example, monokaryons were able to colonise the host as effectively as the parental dikaryon, with three strains even exhibiting a higher percentage of ECM than their parental progenitors. In the absence of the ECM fungus, the poplar root system developed well, as indicated by the control sample, and when interacting with *P. microcarpus* strains, the number of lateral roots decreased in most cases, suggesting that the fungal partner induced a reduction in root biomass through mycorrhization signalling pathways (Fig. 1c). However, this mechanism probably occurred before mycorrhization, as some of the poplar samples that did not have a high number of lateral roots did not form mycorrhizae with the inoculated strain (e.g. in M060, but not in M091; Fig. 1c). In addition, our experiment showed that some strains successfully colonised poplar roots, but did not form a dense hyphal sheath (e.g. in M070 or M118; Fig. 1c,d). Overall, these phenotypic continua point to an underlying genetic basis for the quantitative ECM traits and thus the ability to form mycorrhizas with the root system of the poplar clone.

### Extensive genotypic variation through recombination events

**Fig. 2:**
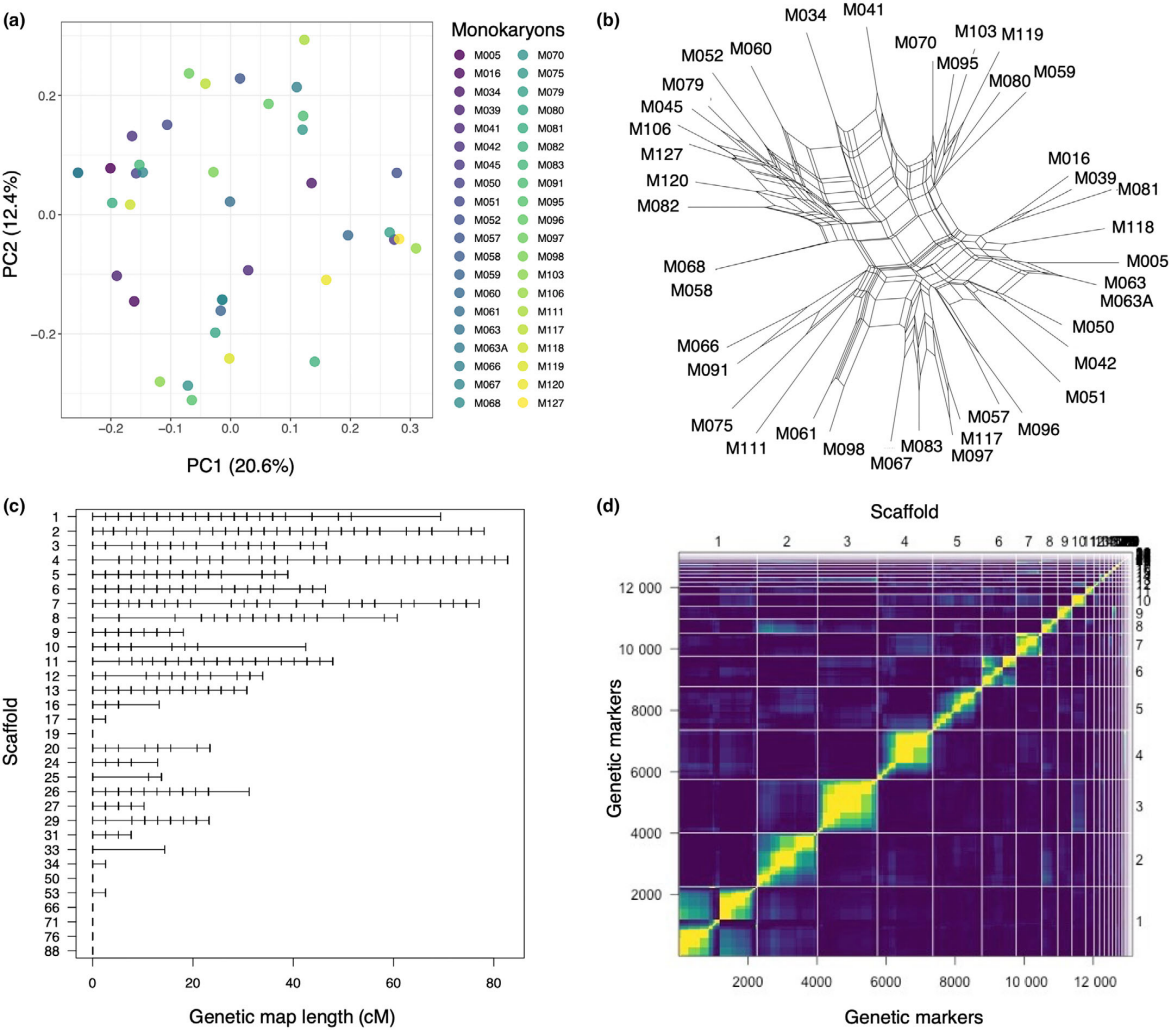
Genotypic variation, genetic map, and linkage groups identified in *Pisolithus microcarpus* sibling monokaryons. (a) Principal component and (b) Neighbor‐Net network analyses based on genome‐wide SNPs at a 1‐kb distance. (c) Genetic map length per scaffold in cM, with the vertical ticks showing the known position of the genetic markers based on the reference genome in the genetic map. (d) Pairwise linkage comparison of markers for the sibling monokaryons. The upper left triangle shows pairwise recombination fractions, and the lower right triangle represents the logarithm of the odds (LOD) scores for tests with *r* = ½. The yellow colour corresponds to a small recombination fraction or a large LOD score, indicating linkage, whereas the dark purple corresponds to the opposite, denoting no linkage. Scaffold identifiers are shown at the top of the graph.

We analysed 13 140 genome‐wide SNPs in 40 *P. microcarpus* sibling monokaryons relative to their parental dikaryon. We used PCA to investigate the genetic relatedness between sibling monokaryons and found a large genotypic variation without discernible genetic clusters along the first PCs (Figs 2a, S4). We inferred a Neighbor‐Net network to identify new allele combinations and detected extensive events of genetic recombination between sibling monokaryons (*P* < 0.001; Figs 2b, S5). The relative positions of the strains in the network analysis are visually consistent with those observed in the ordination analysis.

We constructed a genetic map following the order of the known markers from the physical positions of the SNPs based on the reference genome sequence, and found consistent patterns between the physical and genetic distances of the markers (Figs 2c, S6). The number of SNPs genotyped far exceeded the number of positions on the genetic map, so the SNP markers saturated the number of positions on the genetic map (Figs 2c, S6). No marker exceeded the threshold of 4 for the LOD score, indicating random genotyping errors, and the proportion of missing genotypes along the scaffolds was low, as shown by entropy values below 0.2 (Fig. S6). The total genetic map constructed for the sibling monokaryons spanned 830.90 cM, with a number of SNPs ranging from 5 to 1726 for scaffolds 19 and 1, respectively (Table 1). We found a median genetic distance of 18.08 cM among the scaffolds, with scaffold 4 having the largest length at 82.76 cM. Recombination rates varied between scaffolds irrespective of their marker density or genetic length (Table 1).

**Table 1:** Genetic map summary for *Pisolithus microcarpus* sibling monokaryons.

Scaffold	Start position	End position	Physical length (kb)	Genetic map length (cM)	Recombination rate (cM/Mb)	Number of SNPs
1	570 196	5016 563	4446.37	69.40	15.61	1726
2	30 065	3958 328	3928.26	78.06	19.87	1592
3	114 740	2731 074	2616.33	46.58	17.80	1709
4	33 314	2979 551	2946.24	82.76	28.09	1420
5	218 871	2710 265	2491.39	38.90	15.61	770
6	106 304	2609 755	2503.45	46.44	18.55	1181
7	227 616	2209 983	1982.37	77.07	38.88	552
8	224 531	2415 081	2190.55	60.73	27.73	515
9	520 511	2149 156	1628.65	18.08	11.10	404
10	22 255	1876 054	1853.80	42.49	22.92	144
11	10 340	1831 044	1820.70	47.87	26.29	1038
12	721 159	1557 602	836.44	33.91	40.55	124
13	88 874	1517 266	1428.39	30.78	21.55	613
16	136 561	1041 870	905.31	13.26	14.64	110
17	962 266	1064 330	102.06	2.56	25.13	40
19	272 902	278 975	6.07	0.00	0.00	5
20	66 846	1076 588	1009.74	23.39	23.17	40
24	308 076	904 071	596.00	12.96	21.75	39
25	45 368	475 758	430.39	13.72	31.88	33
26	35 521	491 456	455.94	31.21	68.45	171
27	15 411	614 232	598.82	10.26	17.13	376
29	128 547	722 161	593.61	23.22	39.12	247
31	130 409	702 502	572.09	7.70	13.45	48
33	82	652 754	652.67	14.38	22.04	18
34	443 248	649 145	205.90	2.56	12.46	14
50	22 592	325 015	302.42	0.00	0.00	89
53	44 879	274 598	229.72	2.56	11.16	18
66	123	262 576	262.45	0.00	0.00	33
71	61 488	206 910	145.42	0.00	0.00	7
76	53 777	125 916	72.14	0.00	0.00	28
88	30 475	79 889	49.41	0.00	0.00	36
All			37 863.1	830.90		13 140

We inferred the linkage map using pairwise recombination fractions and retrieved 31 groups (Fig. 2d). We found a distorted diagonal (yellow line) for scaffolds 1 and 6, indicating inconsistencies in the order of SNP markers, although no allele was flagged in the genetic map analysis. These inflated recombination fractions also appeared with lower minimum LOD values on scaffolds 1 and 6 (i.e. 6–7).

### Genomic regions associated with ectomycorrhiza traits

**Fig. 3:**
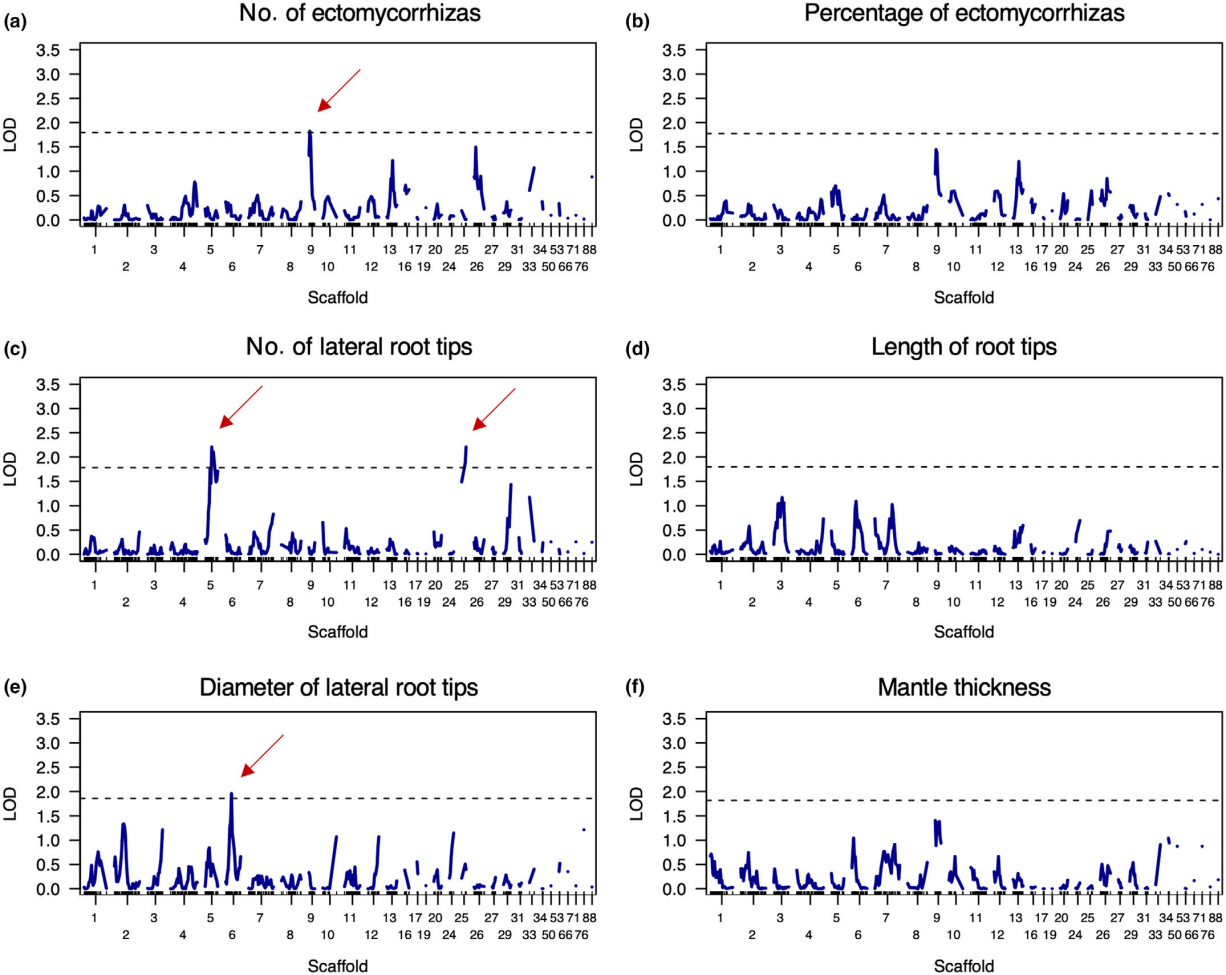
Quantitative trait loci (QTL) mapping for each ectomycorrhizal (ECM) trait in *Pisolithus microcarpus* sibling monokaryons. (a–f) Logarithm of the odds (LOD) scores representing the association for each of the six ECM traits with genomic regions over all scaffolds. The dashed horizontal black line represents the decisive threshold derived from the permutation test, and the dark red arrows show putative QTLs above the defined threshold.

To investigate the genetic basis of ECM traits, we carried out a QTL analysis using simple interval mapping and found four main genomic regions putatively associated with three ECM traits: the number of ectomycorrhizas, the number of lateral root tips, and root tip diameter (Figs 3, S7). For the number of ECMs, we identified one putative QTL on scaffolds 9 and detected another genomic region on scaffold 26 that was weakly associated with the trait (Fig. 3a). The genomic regions on scaffold 9 were also weakly associated with the percentage of ECMs (Fig. 3b), which was consistent with the level of correlation between these two traits (Fig. S2). For the number of lateral root tips, we observed two putative QTL on scaffolds 5 and 25 and found two other genomic regions on scaffolds 29 and 33 that were weakly associated with the trait (Fig. 3c). For the length of root tips, we found a very weak signal in genomic regions localised on scaffolds 3, 6 and 7 (Fig. 3d). For the root tip diameter, we identified a putative QTL on scaffold 6 and recorded several genomic regions weakly associated with the trait on scaffolds 2, 3, 10, 12 and 24 (Fig. 3e). For mantle thickness, we observed the strongest signal on scaffold 9 (Fig. 3f), which coincided with the genomic region associated with the number of ECMs, and to a lesser extent, the percentage of ECMs.

**Fig. 4:**
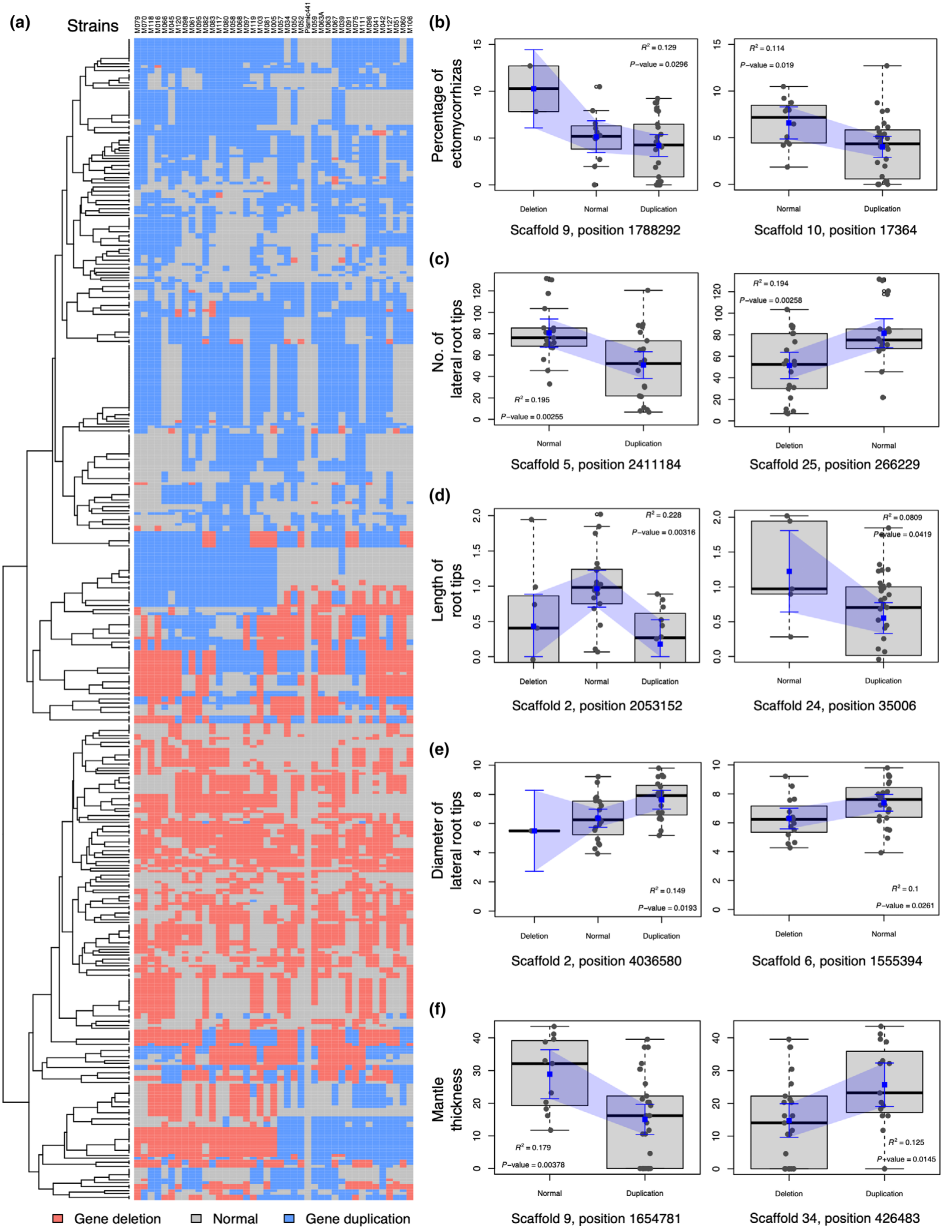
Copy‐number variation (CNV) regions identified in *Pisolithus microcarpus* sibling monokaryons and their associations with ectomycorrhizal (ECM) traits. (a) Heatmap of CNV regions indicating genes affected by deletions or duplications in monokaryons. Tree topology shows grouping according to gene CNV regions. Note that strain 441 displayed only single‐copy genes because the CNV calls were filtered to contain only those genes that were confirmed as single‐copy from the reference genome. The strain codes used are listed in Table S2. (b–f) Generalised additive models of significantly associated CNV regions with ECM traits (*P* < 0.05). Box plots represent the distribution of phenotypic variation in ECM traits across the three different CNV states (Deletion, Normal and Duplication). The blue squares show the model‐predicted means with 95% confidence intervals.

To further assess genetic variation among sibling monokaryons, we focused on structural variants that exhibited a dynamic pattern of genome rearrangement through CNV. To do this, we assembled the genome of each monokaryon strain *de novo* and performed CNV detection by comparing the read depth of the sequencing sets with that of the *P. microcarpus* 441 reference genome (Tables S3, S4). Overall, we found in our studied strains that gene deletions were less frequent than gene duplications and identified 124 and 203 gene deletions and duplications, respectively, by applying a threshold of 50% similarity (Fig. S8). Based on the tree topology of CNV genes, we observed four main clades: (i) a mixed group of gene deletions and duplications, (ii) a pure group of gene deletions, (iii) a mixed group of gene deletions and duplications, and (iv) a group dominated by gene duplications (from bottom to top in Fig. 4a).

We computed GAMs and identified 114 significant associations between CNV regions and ECM traits, including five ECM traits: percentage of ECMs, number of lateral root tips, length of root tips, root tip diameter and mantle thickness (*P* < 0.05; Fig. 4b–f). The highest and lowest numbers of significant associations were observed for the number of lateral root tips (39) and length of root tips (3), respectively (Table S5). No significant association was detected between CNV regions and the number of ectomycorrhizas. The second highest number of associations was found for mantle thickness (36), which also had the highest mean *R* ^2^ among the significant associations, indicating a strong influence of CNV on this trait (Table S5). Across all ECM traits, the mean *R* ^2^ values for the GAM ranged from 0.10 to 0.16, with the highest explanatory power observed for root tip length (*R* ^2^ = 0.23), followed by the number of lateral root tips (*R* ^2^ = 0.20; Table S5).

### Symbiosis‐regulated genes in *Pisolithus microcarpus* strains and poplar clone

**Fig. 5:**
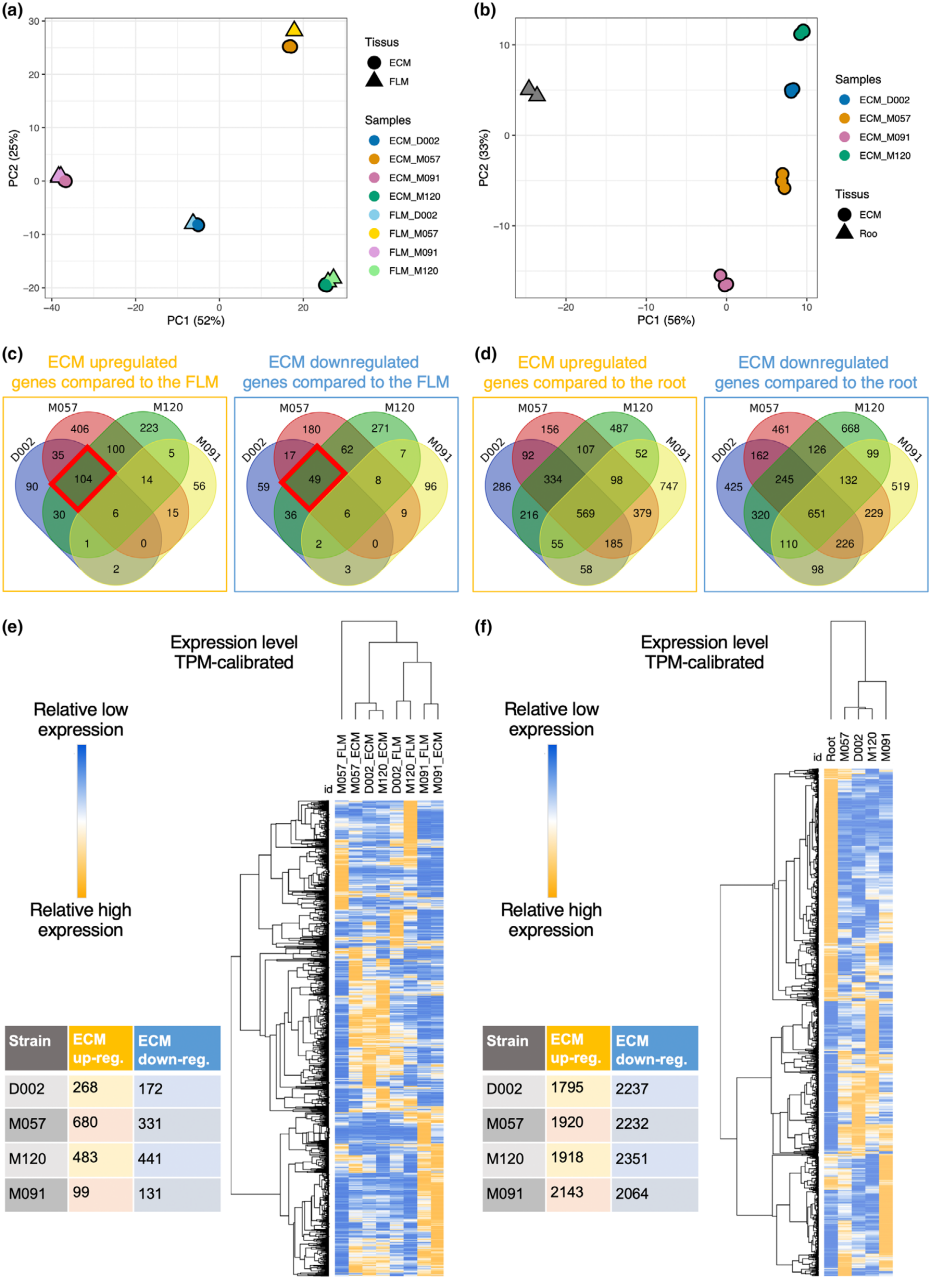
Patterns of gene regulation between ectomycorrhizal (ECM) and free‐living mycelium (FLM) tissues of *Pisolithus microcarpus* strains and between ECM tissues of the poplar clone. (a) Overall transcriptome profiles of ECM and FLM tissues of *P. microcarpus* strains based on principal component analysis (PCA). The percentage values noted in the axis labels refer to the variance explained for each principal component (PC). Colours and symbols indicate strains and tissues (ECM and FLM), respectively (Supporting Information Table S2). (b) Transcriptomic profiles of ECM tissues of poplar clones based on PCA. Colours correspond to strains that form ECMs with poplar clones. (c) Comparison of up‐ and downregulated gene expression in ECM tissues relative to the FLM of *P. microcarpus* monokaryons and the parental dikaryon. Venn diagrams show the overlap of significantly regulated genes for all possible combinations. Red rectangles indicate significantly up‐ and downregulated genes by the three *P. microcarpus* strains capable of forming mycorrhizas with the poplar clone. (d) Comparison of up‐ and downregulated gene expression in ECM tissues of poplar clones relative to the root. (e) Hierarchical clustering analysis of genes significantly regulated in ECM and FLM tissues of *P. microcarpus* strains. (f) Hierarchical clustering analysis of genes significantly regulated in ECM tissues of poplar clones.

To investigate gene regulation mediated by symbiotic interactions, we performed transcriptomic analysis of four *P. microcarpus* strains and the poplar clone, with three replicates each. On the fungal side, we found that differences in gene expression between the monokaryon strains were greater than those between the fungal mycelia; that is, between the ECM and FLM hyphae (Fig. 5a). From the host tree perspective, the results showed a similar pattern to the transcriptomic profile induced by the M091 strain, which differentiated along the first principal component, as expected, because it was unable to form ECMs with the poplar clone (Fig. 5b).

Next, we identified strain‐specific interactions with the poplar clone based on the significantly upregulated and downregulated genes in the ECM tissues compared to those in the FLM (Fig. 5c). In the fungus, the M091 and M057 strains had the lowest (99) and highest (680) numbers of upregulated genes, respectively (Fig. 5c). This ordering was distinct for downregulated genes, with the lowest (131) and highest (441) numbers for the M091 and M120 strains, respectively. For shared interactions, 104 and 49 genes were significantly up‐ and downregulated, respectively, by the three *P. microcarpus* strains capable of forming mycorrhizas with the poplar clone (red rectangles in Fig. 5c). When looking at DEGs between the ECM and FLM, we found a high congruence of up‐ and downregulated genes for the M091 strain, irrespective of contact with the poplar host (Fig. S9).

From the host tree, even though poplar consisted of a single genotype, we also found a high proportion of strain‐specific interactions in the root tissues, with a range of 1795–2143 and 2064–2351 for up‐ and downregulated genes, respectively (Fig. 5d). This suggests that ECM fungal strains elicited major transcriptomic responses in the host symbiont, although a large proportion of interactions were shared between all analysed strains; that is, 569 and 651 genes were significantly up‐ and downregulated, respectively (Fig. 5d). Furthermore, even though it was not possible to form ECMs, direct contact with the fungal strain M091 induced significant up‐ and downregulation of genes in poplar, suggesting that early recognition mechanisms substantially affect the host tree transcriptomic profile (Fig. 5d).

Based on a double hierarchical clustering of significantly regulated genes, two functional groups emerged, corresponding to the tissues of the fungal strains ECM and FLM, with the non‐ECM strain M091 clustered in the FLM group (Fig. 5e). Thus, a gene block shared by the ECM strains exhibited a unique pattern of high relative expression (TPM‐calibrated; Fig. 5e). As for the host, we observed a mirrored clustering pattern of strains, with D002, M057 and M120 grouping together, whereas M091 and the fungus‐free root samples were distinct (Fig. 5f). Regarding gene clustering, we found a large set of genes that were downregulated in the fungus‐free root and M091 but upregulated in the three ECM strains.

To explore the transcriptomic responses of poplar roots interacting with different symbiont genotypes, we analysed gene expression in sibling monokaryons using the parental dikaryon as a reference, excluding fungus‐free root samples. PCA revealed that the gene expression profile of poplar roots interacting with the non‐ECM strain M091 was the most distinct among the sibling monokaryons (Fig. S10a). The parental dikaryon was positioned between the two monokaryons, M057 and M120, along the first two PCs, which together explained 90.0% of the total variance. Overall, we observed the highest number of up‐ and downregulated genes (1067 and 692, respectively) in poplar roots interacting with M091 (Fig. S10b). The number of strain‐specific DEGs in the root tissues ranged from 101 to 756 for upregulated genes and 46 to 627 for downregulated genes (Fig. S10c). The two ECM‐forming strains, M057 and M120, shared 16 upregulated and 8 downregulated genes, respectively. Hierarchical clustering of significantly regulated genes showed well‐defined cluster grouping of ECM‐forming strains (D002, M057 and M120), whereas many DEGs in poplar roots were specific to interactions with the non‐ECM strain M091 (Fig. S10d). Notably, substantial gene expression differences were observed between poplar roots interacting with ECM‐forming strains, supporting the polygenic basis of the continuum of mycorrhization formation.

We then investigated the functional annotation of DEGs putatively involved in symbiosis and found 65 significantly regulated genes in *P. microcarpus* strains D002, M057 and M120 compared to their respective FLM tissues (Table S6). Of these, we found 39 with a putative function with particularly high expression levels, including four SSPs and six CAZymes (Table 2). Several proteins, such as the 32 kDa cell wall symbiosis‐regulated acidic polypeptide precursor and SSPs, are likely involved in modifying the plant cell wall and facilitating fungal colonisation, whereas laccases (AA1_1 enzymes) contribute to lignin degradation and cell wall remodelling, which may support fungal penetration. Oligopeptide and sugar/inositol transporters enable nutrient exchange between symbiotic partners. Glycoside hydrolase family proteins, such as GH27 and GH17, break down polysaccharides and provide accessible sugars to the fungal symbiont. The presence of a GH3 auxin‐responsive promoter suggests a role in plant hormone regulation, which could influence root‐fungus communication.

**Table 2:** Functional annotation of selected differentially expressed genes in ECM tissues of *Pisolithus microcarpus* strains D002, M057 and M120 compared to their respective FLM tissues.

Protein ID	Scaffold ID	Gene start	Gene end	Putative function	Annotation	D002 FLM	D002 ECM	M057 FLM	M057 ECM	M091 FLM	M091 Contact	M120 FLM	M120 ECM
6508	3	107 616	108 496	SSP, 32 kDa‐cell wall symbiosis regulated acidic polypeptide precursor	NCBI Blast, SignalP	89	7025	37	3712	17	25	26	6060
14746	20	106 500	108 610	Cytochrome P450	Interpro	142	682	16	478	16	35	66	699
22374	5	984 826	986 173	Major intrinsic protein	Interpro	40	271	34	124	23	31	43	159
28092	5	553 864	555 559	Glycoside Hydrolase Family 71 protein	CAZy	153	296	79	417	175	108	94	410
92191	6	833 060	835 076	Aldehyde/histidinol dehydrogenase	Interpro	72	297	57	390	61	45	105	480
104664	10	1685 895	1686 599	Phosphatidylethanolamine‐binding protein PEBP	Interpro	10	26	1	8	1	0	14	74
106234	16	522 912	523 721	Acyl‐CoA N‐acyltransferase	Interpro	14	50	9	29	21	31	9	25
111991	8	2348 182	2349 684	Flavonol reductase/cinnamoyl‐CoA reductase	KOG	12	45	18	82	9	10	22	155
119742	16	146 937	149 021	Sugar/inositol transporter	Interpro	60	568	19	322	25	49	49	618
171565	1	1275 726	1276 541	Protein of unknown function DUF3328	Interpro	21	82	57	188	14	21	30	92
212315	29	411 270	414 364	Vacuolar sorting protein VPS1, dynamin	KOG	16	193	15	166	9	12	12	84
373909	11	845 283	847 892	Zn(2)‐C6 fungal‐type DNA‐binding domain	Interpro	82	250	82	516	122	117	68	276
440048	3	110 513	112 243	32 kDa‐cell wall symbiosis regulated acidic polypeptide precursor	NCBI Blast	175	15 871	146	10 491	22	36	45	15 404
466494	12	1044 394	1044 935	SSP	SignalP	12	24	12	38	11	18	3	8
481168	27	175 068	177 539	AA1_1, Laccase	CAZy	11	81	6	107	9	18	4	136
573514	29	169 193	170 680	Carbonic anhydrase	Interpro	44	210	15	83	20	22	14	165
670385	1	2347 962	2351 545	Oligopeptide transporter	Interpro	55	234	17	326	15	19	5	127
672167	27	281 358	286 627	Zn(2)‐C6 fungal‐type DNA‐binding domain	Interpro	35	144	13	86	45	88	17	115
674729	5	256 492	258 922	GH3 auxin‐responsive promoter	Interpro	32	106	28	111	54	37	20	139
676173	6	1679 812	1683 139	Zn(2)‐C6 fungal‐type DNA‐binding domain	Interpro	28	67	21	64	23	25	18	52
677400	9	784 122	786 832	Carbohydrate Esterase Family 4 protein	Cazy	51	123	32	118	10	15	38	137
685962	4	998 676	1001 279	Cytochrome P450	Interpro	52	112	20	146	37	39	25	119
1189951	10	497 825	501 267	Glycoside Hydrolase Family 27 protein	CAZy	2	13	5	24	5	5	0	1
2602805	4	440 958	442 661	Archaeal/bacterial/fungal rhodopsin‐like	Interpro	56	154	30	233	45	46	24	117
2604015	4	451 776	452 263	SSP	SignalP	36	164	4	79	5	3	68	194
3369665	7	2147 882	2149 942	Phophatidylserine decarboxylase	Interpro	21	134	26	154	4	4	6	16
4111547	29	104 875	105 506	HSP20‐like chaperone	Interpro	225	482	31	305	0	1	328	1143
4127034	13	780 603	781 124	SSP	SignalP	1	6	0	19	1	2	1	14
4127905	17	44 852	46 090	Zinc‐binding oxidoreductase	KOG	9	45	9	26	6	7	8	61
4166157	1	2279 962	2282 359	Glutamate decarboxylase	Interpro	46	98	10	97	33	44	40	113
4180914	2	1344 097	1347 166	Cytochrome P450	Interpro	46	95	22	88	32	42	34	140
4241248	6	513 633	515 804	Major facilitator superfamily domain	Interpro	72	176	23	156	61	89	58	312
4245467	6	1378 171	1380 747	Proteins containing the FAD binding domain	KOG	72	159	31	188	80	132	46	107
4282329	11	588 100	589 105	FMN‐binding split barrel	Interpro	38	183	13	61	8	6	63	174
4282581	11	631 344	632 244	Cation efflux protein	Interpro	11	76	6	33	20	36	7	56
4293235	12	1412 262	1413 177	Phosphatidylethanolamine‐binding protein PEBP	Interpro	8	21	2	14	4	3	4	23
4357186	31	66 431	68 842	Cytochrome P450	KOG	52	207	6	161	7	11	28	236
4464565	13	186 456	188 388	Metalloexopeptidases	KOG	437	1543	34	1212	20	30	86	980
4493123	9	1065 700	1067 122	Glycoside Hydrolase Family 17 protein	CAZy	25	117	19	119	32	44	19	124

The upregulated genes also identified as upregulated in M09,1 were excluded to restrict the top‐candidate genes to symbiosis‐related interactions. A complete list of DEGs is presented in Supporting Information Table S6.

### Balanced distribution of gene variants in monokaryon progeny at mating type loci

**Fig. 6:**
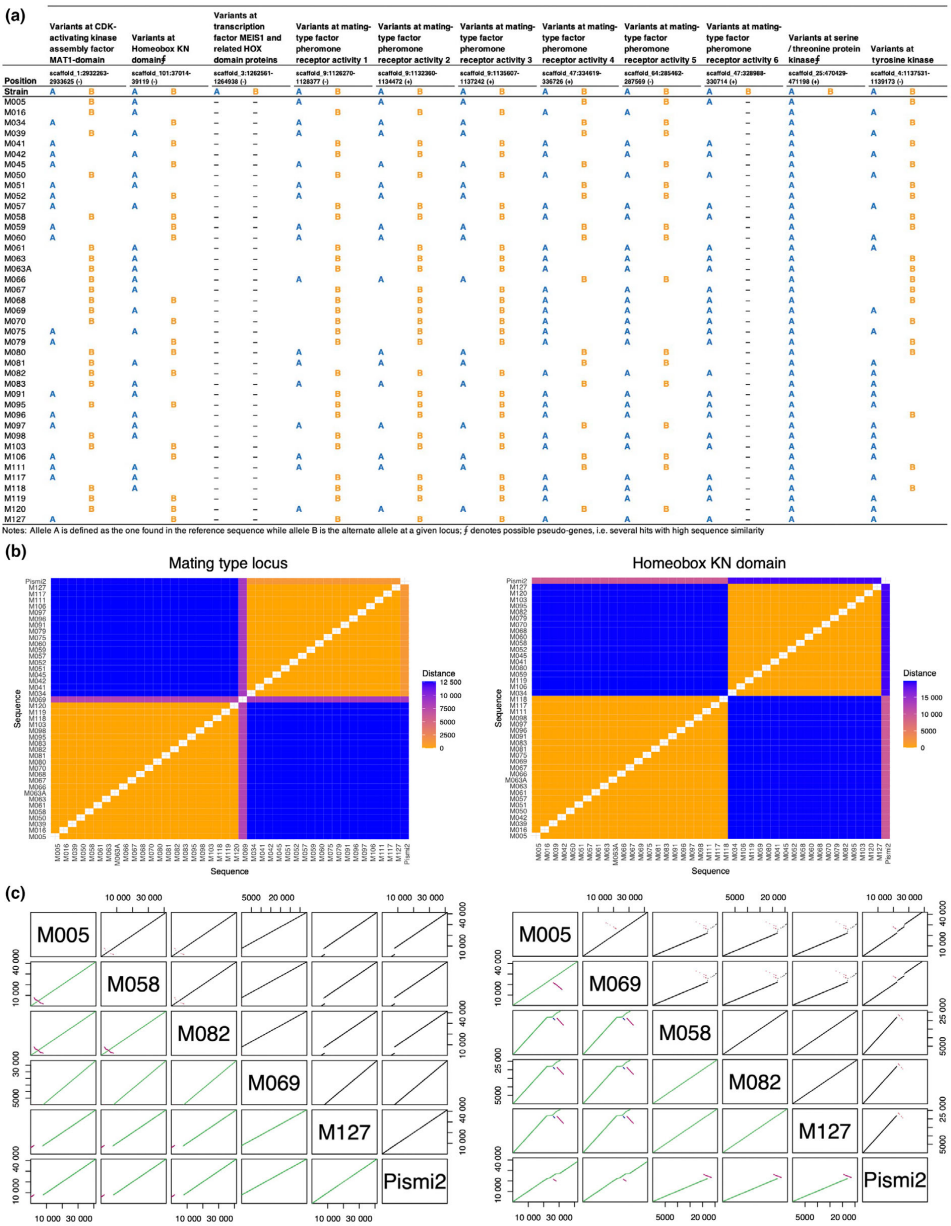
Allele segregation and evidence of recombination at 11 mating‐type loci in the *Pisolithus microcarpus* sibling monokaryons. (a) Distribution of allele variants at 11 mating‐type‐related loci among monokaryons. Blue and orange represent the sequences of the reference and alternative alleles, respectively (A and B). Reference idiomorphs were defined as those found in the reference strain, *P. microcarpus* 441. (b) Pairwise genetic distances of monokaryons for two selected genes involved in mating type, including the reference strain *P. microcarpus* 441. Orange and blue represent low and high genetic distances, respectively. (c) Synteny analysis of 20 kbp upstream and downstream of mating‐type loci. Five selected monokaryons were represented alongside the reference strain 441. Syntenic hits are shown in the upper left, while syntenic blocks are shown in the bottom left. In the upper triangle, hits in black indicate collinearity, whereas hits in magenta represent inverted regions. In the bottom triangle, the blocks are coloured by score.

To better understand sexual reproduction in *P. microcarpus*, we analysed gene variants at 11 mating‐type loci to assess how allele segregation occurred in monokaryons during meiosis from their parental dikaryon, and to what extent recombination generates new allele combinations among siblings. These multi‐allelic mating‐type loci are known to be involved in the cell‐specific identity determinant, the specialised kinase cascade that controls mating, and in proteins encoding pheromones and pheromone receptors (de Freitas Pereira *et al*., 2017). We used reference sequences from the mating‐type loci characterised in the reference genome assembly (strain 441 v.2; Plett *et al*., 2023) and retrieved two allelic variants from our *de novo* monokaryon genome assemblies at eight loci (Fig. 6a).

Overall, we found a balanced distribution of these alleles in the progeny monokaryons, as expected from random allele sorting during meiosis (Figs 6a, S11). As shown in several basidiomycetes (Niculita‐Hirzel *et al*., 2008), the gene encoding the *MAT1* domain of the CDK activating kinase assembly factor was highly conserved, with the two alleles differing by only 13 bp, with no segregating sites within these two variants (Fig. 6b). Interestingly, multilocus genotypes were observed among the monokaryons, supporting recombination events between these genomic regions. However, the five pheromone‐like receptor activity genes were strongly linked, although they were located in three different scaffolds of the reference genome assembly (Fig. 6a). A single allele of the factor pheromone receptor activity 6 was consistently found in half of the monokaryons with 100% similarity to the reference sequence, while no alternative allele was found in the other strains. In addition, the gene encoding the serine–threonine protein kinase was monomorphic (Fig. 6a), but a BLAST search identified two putative pseudogenes with greater than 90% similarity to the single reference sequence. Together, these results confirm the heterothallism and tetrapolar system of the species and highlight that recombination may or may not contribute to allele combinations in progeny, depending on the different mating type loci.

Upon examining the 20 kbp upstream and downstream flanking regions of the CDK activating kinase assembly factor and the Homeobox KN domain, we observed the presence of synteny blocks indicative of genomic inversions (Fig. 6c). These inversions were substantial between several pairwise sibling monokaryons such as M005 and M058. By contrast, other strains, such as M127, exhibited a high degree of synteny with reference genome 441, showing no signs of genomic rearrangement (Fig. 6c). Even though sequence similarity for each idiomorph of these two mating‐type‐related loci remains remarkably high (Fig. 6b), we identified subtle variations in the genomic architecture that suggest divergence among the strains, inherited from the parental progenitors, or being the product of recombination. These differences may provide insights into the underlying mechanisms of genomic stability and variation in mating‐type regions across both closely related isolates (sibling monokaryons) and distantly related isolates (monokaryons compared to the reference strain 441).

## Discussion

Our study demonstrates that symbiotic trait variation among sibling monokaryons of *P. microcarpus* is shaped by genetic recombination and divergent transcriptomic responses. Using phenotypic, genomic and transcriptomic data, we identified broad phenotypic clines for ECM traits along with putative QTL and CNV regions linked to ECM trait variation. Additionally, we observed strain‐specific transcriptomic responses in both symbiont and host tissues. Phenotypic analysis revealed significant differences in ECM traits, with some sibling monokaryons forming well‐developed ECM associations, whereas others showed minimal or no colonisation of the host tree (Fig. 1). This suggests that ECM trait formation is influenced by multiple genetic factors and is likely governed by quantitative genetic variation resulting from recombination events among monokaryons (Fig. 2). We also identified putative QTL and CNV regions associated with ECM traits, highlighting candidate genomic regions underlying symbiotic compatibility (Figs 3, 4). In parallel, we uncovered distinct gene expression patterns between ECM and FLM mycelia along with strain‐specific host responses (Fig. 5). These findings underscore the dynamic nature of intraspecific fungal transcriptomes during symbiosis, with gene regulation fine‐tuned by host‐fungus genotype interactions. Furthermore, our results highlight the role of mating‐type recombination in shaping genetic diversity, confirming the heterothallic, tetrapolar system of *P. microcarpus* and revealing genomic rearrangements in the mating‐type loci flanking regions among sibling monokaryons (Fig. 6).

### Ectomycorrhizal trait variation at intraspecific level

Here, we show substantial intraspecific phenotypic variation in ECM traits among *P. microcarpus* sibling monokaryons, which varied in their ability to form ECM associations with poplar. Notably, some monokaryons demonstrated superior colonisation rates compared with the parental dikaryon (Fig. 1), highlighting the potential for genetic recombination to produce progeny with enhanced symbiotic abilities. This intraspecific phenotypic variation aligns with previous studies that demonstrated that ECM trait expression is shaped by both genetic and environmental factors (Rosado *et al*., 1994b; Silva *et al*., 2007). Previous studies have revealed that the degree of phenotypic variation extends across several key ECM traits, including the percentage of root tips colonised, mantle thickness and lateral root development (Burgess *et al*., 1994; Costa *et al*., 2010). The observed phenotypic continuum from incompatible to fully compatible strains suggests that ECM formation is a complex quantitative trait that is likely controlled by multiple genetic loci and influenced by epistatic interactions.

From a practical perspective, intraspecific variability in ECM formation has significant implications for the use of fungal inoculants. The observed phenotypic variation among monokaryons suggests that spore‐based inoculants, which rely on random mating, may produce inconsistent outcomes owing to the recombination of unfavourable alleles (Rosado *et al*., 1994b; Costa *et al*., 2010). This is particularly important when considering quantitative traits, as random crosses between monokaryons may not lead to predictable outcomes. For example, two monokaryons with unfavourable alleles may cross, resulting in progeny that perform poorly in terms of ECM formation. By contrast, controlled pairing of monokaryons with desirable traits could improve inoculant reliability, leading to more predictable ECM establishment and enhanced tree growth. This approach offers a more effective strategy for selecting strains with optimised traits for forestry applications, as it takes advantage of known recombination patterns to direct crosses between monokaryons that would yield enhanced quantitative traits. Understanding the specific alleles involved in ECM formation can further refine the selection strategies for the development of high‐performance fungal inoculants. Future research should focus on refining these strategies, identifying optimal monokaryon strains, and exploring other quantitative traits such as drought resistance and nutrient uptake efficiency, which could complement the broader goals of enhancing forest productivity and resilience (Silva *et al*., 2007; Plett *et al*., 2022).

### Genomic regions involved in ectomycorrhizal fungal traits

Pioneering work by Rosado *et al*. on *P. tinctorius* and its ECM formation in pine trees demonstrated strong inheritance of ECM traits, suggesting that some ECM traits are controlled by multiple genes (Rosado *et al*., 1994a,b). Their research underscored the importance of understanding both the recombination capacity and genetic effects that govern ECM traits, which would allow for strategic crossing of monokaryons to produce progeny with enhanced ECM potential. In this study, we found evidence for genetic recombination among sibling monokaryons (Fig. 2) and identified through QTL and CNV analysis the genomic regions associated with ECM traits, for example, the number of ECMs formed, the number of lateral root tips and the diameter of lateral root tips (Figs 3, 4). The strongest QTL signals were found on scaffolds 5, 6 and 9, whereas the strongest associations with CNV regions were detected on scaffolds 9 and 34 (mean *R* ^2^ = 0.16; Table S5). Our analyses, based on SNPs and SVs, showed that testing traits with different types of genetic markers provided complementary results with common or distinctive associations. For instance, the ECM trait, the number of lateral root tips, showed two putative QTL and 39 significant associations with CNV regions. However, no QTL was detected for mantle thickness, although it was associated with 36 CNV regions.

These genomic regions are likely to harbour candidate genes associated with the main stages of ECM formation, including pre‐contact (Jambois & Lapeyrie, 2005), colonisation (Tagu *et al*., 2002), differentiation (Martin *et al*., 2001) and functioning (Pardo *et al*., 2005). Several genes involved in fungal growth, signalling and nutrient exchange may either enhance or impede symbiotic interactions (Kohler *et al*., 2015; Miyauchi *et al*., 2020). Although we identified genomic regions associated with ECM traits, several factors limited our ability to specify candidate genes and their functional annotations. First, we detected broad putative QTLs that did not reach statistical significance based on a 1000‐permutation test, suggesting that the QTL‐based associations with the traits were modest. Second, the high linkage disequilibrium in the genomic data from sibling monokaryons complicates the distinction between closely linked gene variants underlying these traits. Third, the presumably high genetic relatedness between the progenitors of the parental dikaryon, combined with a modest sample size (i.e. 40 monokaryons), reduced the resolution and statistical power of our QTL analysis, potentially masking subtle genetic effects. Consequently, while these findings provide valuable insights, fine mapping of these putative QTLs is necessary to identify causal genetic variants and validate candidate genes.

### Interplay of host–symbiont transcriptomic responses

Our transcriptomic analysis revealed substantial variations in gene expression among *P. microcarpus* strains, with differences between monokaryons exceeding those between fungal tissues (ECM vs FLM; Fig. 5). This suggests that genetic background plays a more significant role in transcriptional regulation than tissue differentiation, consistent with previous studies on ECM fungi that highlighted strain‐dependent symbiotic capabilities (Plett *et al*., 2021). Notably, the M091 strain, which failed to establish ECM with poplar, exhibited a distinct transcriptomic profile, supporting its functional divergence from ECM‐capable strains (Figs S9, S10). Strain‐specific responses were marked in the number of DEGs, with M057 showing the highest number of up‐ and downregulated genes in ECM tissue and M091 the lowest (Fig. 5e). A core set of genes was consistently up‐ or downregulated in all ECM‐forming strains, indicating a shared molecular basis for symbiosis. Functional annotation of these genes highlighted the involvement of two previously identified symbiosis‐induced 32‐kDa SRAP32s proteins (Laurent *et al*., 1999), an aquaporin (protein ID 22374; Table 2) and six carbohydrate‐active enzymes (CAZymes; Table 2), which are crucial for host colonisation and fungal metabolism (Kohler *et al*., 2015; Miyauchi *et al*., 2020). Hierarchical clustering grouped M091 interacting with poplar roots with the FLM, reinforcing its inability to engage in an ECM state. These findings underscore the genotype‐dependent nature of gene regulation in *P. microcarpus* strains, where genetic variation drives differential symbiotic abilities, and highlight the importance of both conserved and strain‐specific transcriptional programmes in ECM formation.

From a host perspective, poplar exhibited extensive transcriptomic reprogramming in response to different *P. microcarpus* strains, with strain‐specific responses evident even within interactions with sibling monokaryons (Fig. 5f). Analysis of poplar transcriptional responses revealed that the most highly regulated transcripts during successful ECM formation with compatible strains were also commonly induced across ECM‐forming monokaryons, suggesting activation of shared fungal recognition pathways. However, strain‐specific host responses were also evident, including differential regulation of kinases and a gene with homology to DLO1 (DMR6‐LIKE OXYGENASE 1) from Arabidopsis, a negative regulator of defence responses, which was upregulated specifically during compatible interactions. The strain M091, despite failing to form ECM, induced substantial transcriptional changes in poplar roots, suggesting that early recognition events significantly shape host responses regardless of symbiotic outcome (Plett *et al*., 2014). Genes uniquely regulated during M091 interactions included stress and defence‐related transcripts as well as signalling and transporter genes, indicating that the host can molecularly distinguish between compatible and incompatible fungal partners (Fig. S10). Principal component analysis revealed that the poplar roots interacting with ECM strains clustered separately from those exposed to M091, supporting distinct transcriptomic shifts in response to compatible vs incompatible fungal partners (Fig. S10). The parental dikaryon positioned between the two ECM‐forming monokaryons further suggests an additive or intermediate effect of genetic recombination on host responses. These results reinforce the polygenic nature underlying ECM formation and suggest that, while core pathways drive symbiosis, fungal strain‐specific factors modulate the extent and efficiency of host engagement, ultimately shaping the success of mycorrhization.

### Recombination of mating type loci

Genetic recombination plays a crucial role in shaping fungal genomes, contributing to genetic diversity, adaptation and evolution (Croll *et al*., 2015; Gladieux *et al*., 2015; LoBuglio & Taylor, 2017). Our analyses of *P. microcarpus* sibling monokaryons revealed extensive recombination events, as evidenced by the Neighbor‐Net network analysis, and showed the absence of distinct genetic clusters in the PCA (Fig. 2). This suggests a high level of genetic intermixing among progeny, similar to patterns observed in other basidiomycetes, where recombination supports major genomic rearrangements (Heinzelmann *et al*., 2020). Our inferred genetic map showed consistency between physical and genetic distances, indicating a robust framework for analysing recombination events (Fig. 2). This aligns with findings in other fungi, where high‐density linkage maps revealed widespread recombination and crossover events (Forche *et al*., 2008; Wittenberg *et al*., 2009). Although recombination rates varied across scaffolds of *P. microcarpus*, they did not correlate with marker density or genetic length, suggesting region‐specific recombination dynamics (Nieuwenhuis & James, 2016). These results highlight the critical role of recombination in generating novel allele combinations in ECM fungal genomes and emphasise their contribution to the genetic diversity and functional consequences within fungal populations.

Recombination is essential for the evolution and functional dynamics of mating‐type loci in basidiomycetes (Raudaskoski & Kothe, 2010). As expected, we found balanced allele distributions at the mating‐type loci, consistent with random allele segregation during meiosis (Fig. 6). Despite this, recombination generated new multi‐locus genotypes, highlighting its impact on allele assortment at mating‐type loci. However, the five pheromone receptor genes remain strongly linked, even when located across different scaffolds, suggesting evolutionary constraints that maintain their association (Coelho *et al*., 2017). Notably, a serine/threonine kinase displayed monomorphism, with a single allele consistently present in all monokaryons, potentially indicating homozygosity and/or a conserved function that is critical for mating success (Casselton & Kües, 2007). Genomic rearrangements were evident within the mating‐type loci, particularly in the flanking regions of the CDK‐activating kinase assembly factor and the Homeobox KN domain where synteny blocks indicative of genomic inversions were observed. While some sibling monokaryons exhibited substantial inversions, others maintained high synteny with the reference genome of *P. microcarpus* 441, reflecting either differential recombination effects on the genome architecture or an inherited parental polymorphism. These findings provide new insights into the evolutionary forces shaping mating‐type loci in ECM fungi, emphasising the interplay between recombination, genome stability and mating compatibility in fungal populations.

### Conclusions and future perspectives

This study revealed how intraspecific genetic diversity, shaped by recombination among sibling monokaryons, shapes ECM trait variation in *P. microcarpus* during symbiosis with a poplar clone. Through QTL and CNV analyses, we identified key genomic regions associated with ECM traits, supporting the hypothesis that the establishment of symbiosis is a complex, polygenic trait. We further observed strain‐specific fungal transcriptomic responses and host reprogramming, revealing how fungal intraspecific genetic variation induces a continuum from incompatible interactions to fully developed ECM structures. Moreover, our investigation of mating‐type loci confirmed a heterothallic tetrapolar system, highlighting the role of recombination in generating new allele combinations in *P. microcarpus*. These findings deepen our understanding of fungal evolution and symbiosis, with direct implications for optimising fungal inoculants for forestry and ecosystem restoration. Future research should focus on fine‐mapping the candidate genes involved in ECM formation and assessing the ecological fitness of different monokaryon‐dikaryon combinations in natural environments. Additionally, exploring the role of epigenetic modifications in symbiotic regulation may provide further mechanistic insights into fungal mutualism. Integrating functional genomics with experimental ecology is crucial for understanding the evolutionary dynamics of ECM associations and their contribution to forest resilience in changing environments.

## Competing interests

None declared.

## Author contributions

AK, FM and MDC acquired funding. AK, BD, FM, MDC, MP and MFP designed the study. MFP performed laboratory work with the support of AK, FG, LF and TCA. AK, BD and MFP analysed the phenotypic data. BD performed bioinformatics and genomic analyses with the support of DC. AK, BD and MFP analysed the transcriptomic data. BD wrote the manuscript with contributions from AK, FM, MDC, MFP and MP. All authors have read and approved the final version of the manuscript. DB and MFP contributed equally to this work.

## Disclaimer

The New Phytologist Foundation remains neutral with regard to jurisdictional claims in maps and in any institutional affiliations.

## Supporting information


**Fig. S1** Morphological differences between the *Pisolithus microcarpus* parental dikaryon and sibling monokaryons grown in pure culture.
**Fig. S2** Pairwise Pearson's correlation coefficients between ectomycorrhizal traits in *Pisolithus microcarpus* strains.
**Fig. S3** Phenotypic variation in ECM traits in *Pisolithus microcarpus* strains based on an ordination method.
**Fig. S4** Genotypic variation of *Pisolithus microcarpus* sibling monokaryons based on ordination analysis.
**Fig. S5** Distribution of allele frequencies and genotypes among *Pisolithus microcarpus* sibling monokaryons.
**Fig. S6** Position of single nucleotide polymorphisms and proportion of missing genotypes among *Pisolithus microcarpus* sibling monokaryons.
**Fig. S7** Quantile–quantile plots of Box–Cox‐transformed ECM traits among *Pisolithus microcarpus* sibling monokaryons.
**Fig. S8** Gene content including CNV with percentage of length covered by deletion or duplication > 50%.
**Fig. S9** Patterns of gene regulation in *Pisolithus microcarpus* strains in different ECM and FLM tissues.
**Fig. S10** Patterns of gene regulation in Populus roots in contact with *Pisolithus microcarpus* strains.
**Fig. S11** Allele segregating at eight mating type loci in *Pisolithus microcarpus* sibling monokaryons.
**Table S1** Description of the ectomycorrhizal traits analysed in this study.
**Table S2** Phenotypic variation for each *Pisolithus microcarpus* strain analysed in this study.
**Table S3** Summary of genome sequencing data with mapping statistics from the studied *Pisolithus microcarpus* strains.
**Table S4** Characteristics of *Pisolithus microcarpus de novo* genome assemblies analysed in this study.
**Table S5** Number of significant associations between CNV regions and ECM traits from the studied *Pisolithus microcarpus* strains using generalised additive models.

**Table S6** Complete list of upregulated genes in ECM tissues of D002, M057 and M120 compared to their respective FLM tissues.
Please note: Wiley is not responsible for the content or functionality of any Supporting Information supplied by the authors. Any queries (other than missing material) should be directed to the *New Phytologist* Central Office.

## Data Availability

Genomic data were deposited at NCBI under the BioProject PRJNA1274373 (accession nos. SAMN48981554–SAMN48981594). Transcriptomic data were deposited at NCBI under the Gene Expression Omnibus project GSE298025 (accession nos. GSM9004899–GSM9004922). Phenotypic data are presented in Table S2.
