# Supplementary material for: Genetic variation among progeny shapes symbiosis in a basidiomycete with poplar
Source: New Phytol. 2025 Aug 7;248(1):157–77. doi: 10.1111/nph.70395 (PMC12409109; doi:10.1111/nph.70395)
Supplement: Supplementary file 1 — Fig. S1 Morphological differences between the Pisolithus microcarpus parental dikaryon and sibling monokaryons grown in pure culture. Fig. S2 Pairwise Pearson's correlation coefficients between ectomycorrhizal traits in Pisolithus microcarpus strains. Fig. S3 Phenotypic variation in ECM traits in Pisolithus microcarpus strains based on an ordination method. Fig. S4 Genotypic variation of Pisolithus microcarpus sibling monokaryons based on ordination analysis. Fig. S5 Distribution of allele frequencies and genotypes among Pisolithus microcarpus sibling monokaryons. Fig. S6 Position of single nucleotide polymorphisms and proportion of missing genotypes among Pisolithus microcarpus sibling monokaryons. Fig. S7 Quantile–quantile plots of Box–Cox‐transformed ECM traits among Pisolithus microcarpus sibling monokaryons. Fig. S8 Gene content including CNV with percentage of length covered by deletion or duplication > 50%. Fig. S9 Patterns of gene regulation in Pisolithus microcarpus strains in different ECM and FLM tissues. Fig. S10 Patterns of gene regulation in Populus roots in contact with Pisolithus microcarpus strains. Fig. S11 Allele segregating at eight mating type loci in Pisolithus microcarpus sibling monokaryons. Table S1 Description of the ectomycorrhizal traits analysed in this study. Table S2 Phenotypic variation for each Pisolithus microcarpus strain analysed in this study. Table S3 Summary of genome sequencing data with mapping statistics from the studied Pisolithus microcarpus strains. Table S4 Characteristics of Pisolithus microcarpus de novo genome assemblies analysed in this study. Table S5 Number of significant associations between CNV regions and ECM traits from the studied Pisolithus microcarpus strains using generalised additive models. [file NPH-248-157-s001.docx]

## *New Phytologist* Supporting Information

Article title: **Genetic variation among progeny shapes symbiosis in a basidiomycete with poplar**

Authors: Benjamin Dauphin, Maíra de Freitas Pereira, Daniel Croll, Thalita Cardoso Anastácio, Laure Fauchery, Frédéric Guinet, Maurício Dutra Costa, Francis Martin, Martina Peter, Annegret Kohler

Article acceptance date: 25 June 2025


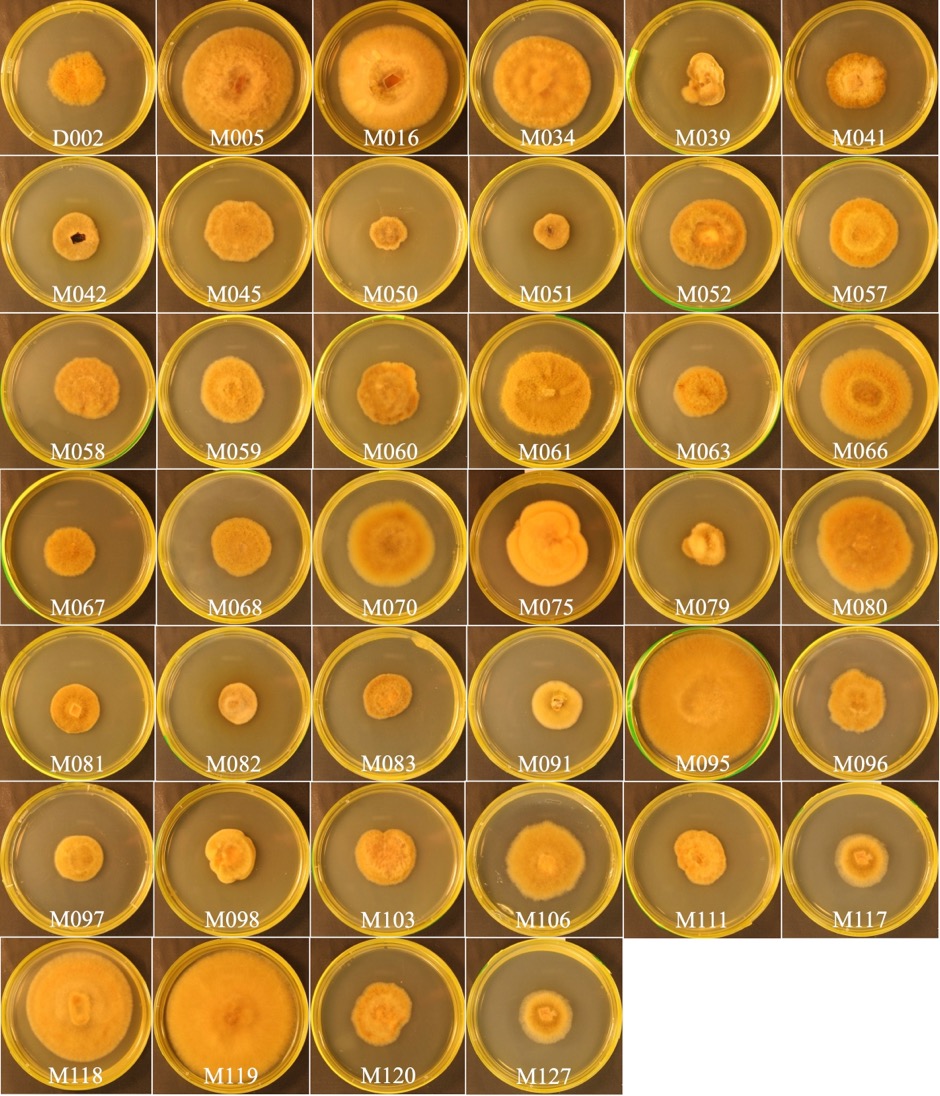


**Fig. S1 Morphological differences between the *Pisolithus microcarpus* parental dikaryon and sibling monokaryons grown in pure culture.**

**Fig. S2 Pairwise Pearson’s correlation coefficients between ectomycorrhizal traits in *Pisolithus microcarpus* strains.** Above the diagonal, more intense colours indicate stronger positive (blue) and negative (red) correlations. The square sizes were proportional to the pairwise correlation values. Pairwise Pearson correlation coefficients are represented below the diagonal. The phenotypic traits and abbreviation names are listed in Table S1.

**Fig. S3 Phenotypic variation in ECM traits in *Pisolithus microcarpus* strains based on an ordination method.** Principal component (PC) analysis showing (**a**-**b**) PC1 and PC2, and (**c**-**d**) PC1 and PC3 for the *P. microcarpus* strains and ECM traits, respectively. The percentage values noted in the axis labels refer to the percentage of total variance explained. Orange and green colours represent dikaryon and monokaryon strains, respectively. The strain codes are listed in Table S2.

(a)

(b)

**Fig. S4 Genotypic variation of *Pisolithus microcarpus* sibling monokaryons based on ordination analysis.** Principal component (PC) analysis showing (**a**–**b**) PC1 and PC2, PC1, and PC3, respectively. Percent values noted in the axis labels refer to the percentage of total variance explained. Colours represent different monokaryon strains. Strain codes are listed in Table S2.


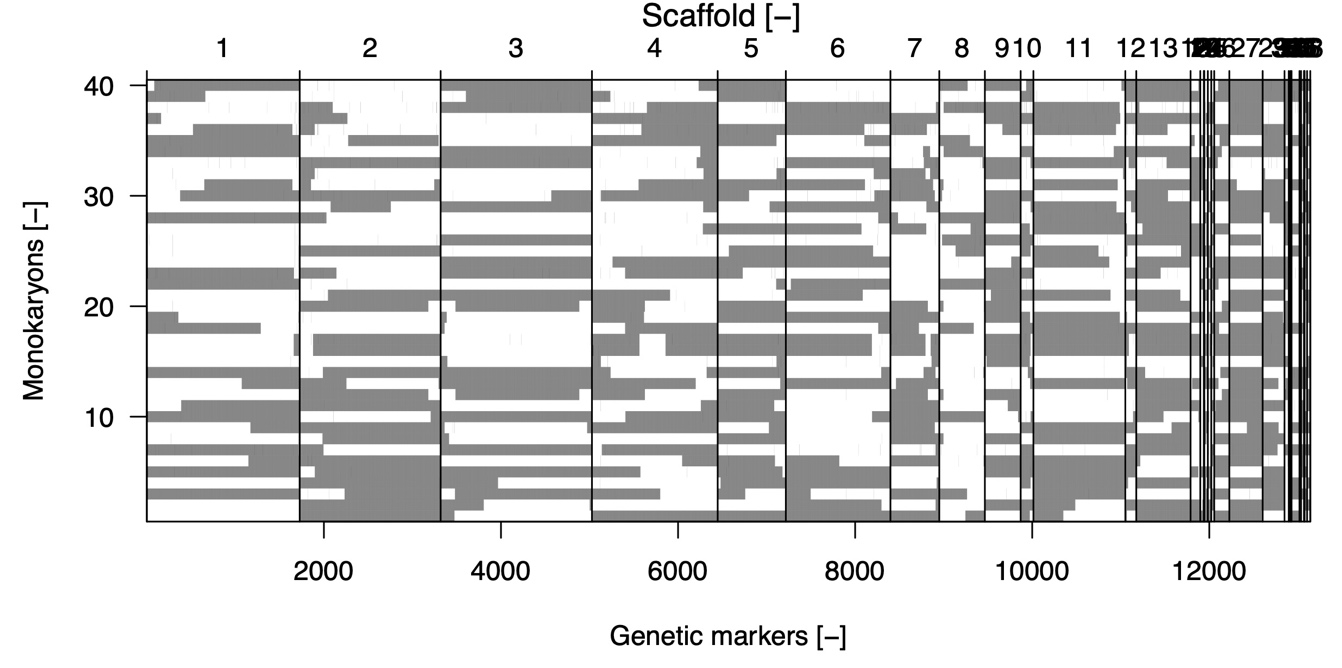


(a)

(b)

**Fig. S5 Distribution of allele frequencies and genotypes among *Pisolithus microcarpus* sibling monokaryons.** (**a**) Distribution of allele frequencies among sibling monokaryons. (**b**) Genotype distribution along scaffolds with monokaryons in rows, indicating crossing over and recombination sites. The colour grey and white represent the “A” an “B” genotype, respectively. Scaffold identifiers are denoted at the top of the graph.

(b)

(a)

**Fig. S6 Position of single nucleotide polymorphisms and proportion of missing genotypes among *Pisolithus microcarpus* sibling monokaryons.** (**a**) Length of scaffolds in bp, with vertical ticks showing the position of genetic markers for each scaffold. (**b**) Proportion of missing information in the genotype dataset using the entropy method. A value of 1 indicates that genotypes are equally likely, and a value of 0 indicates that genotypes are completely determined.

**Fig. S7 Quantile-Quantile (QQ) plots of Box–Cox transformed ECM traits among *Pisolithus microcarpus* sibling monokaryons.** Each panel represents one of the six ECM traits. The normal quantiles on the x-axis were compared with the observed quantiles of each trait on the y-axis to assess the normality of the transformed trait distributions.

**Fig. S8 Gene content including CNV with percentage of length covered by deletion or duplication >50%.** Deletions refer to any gene locus showing at least one deletion in a strain, whereas duplications include any gene locus showing only duplication or normal copy numbers without deletion. CNVs with a minor allele frequency (MAF) of < 0.15. These were filtered out to reduce false positives (see Mat. & Meth.).


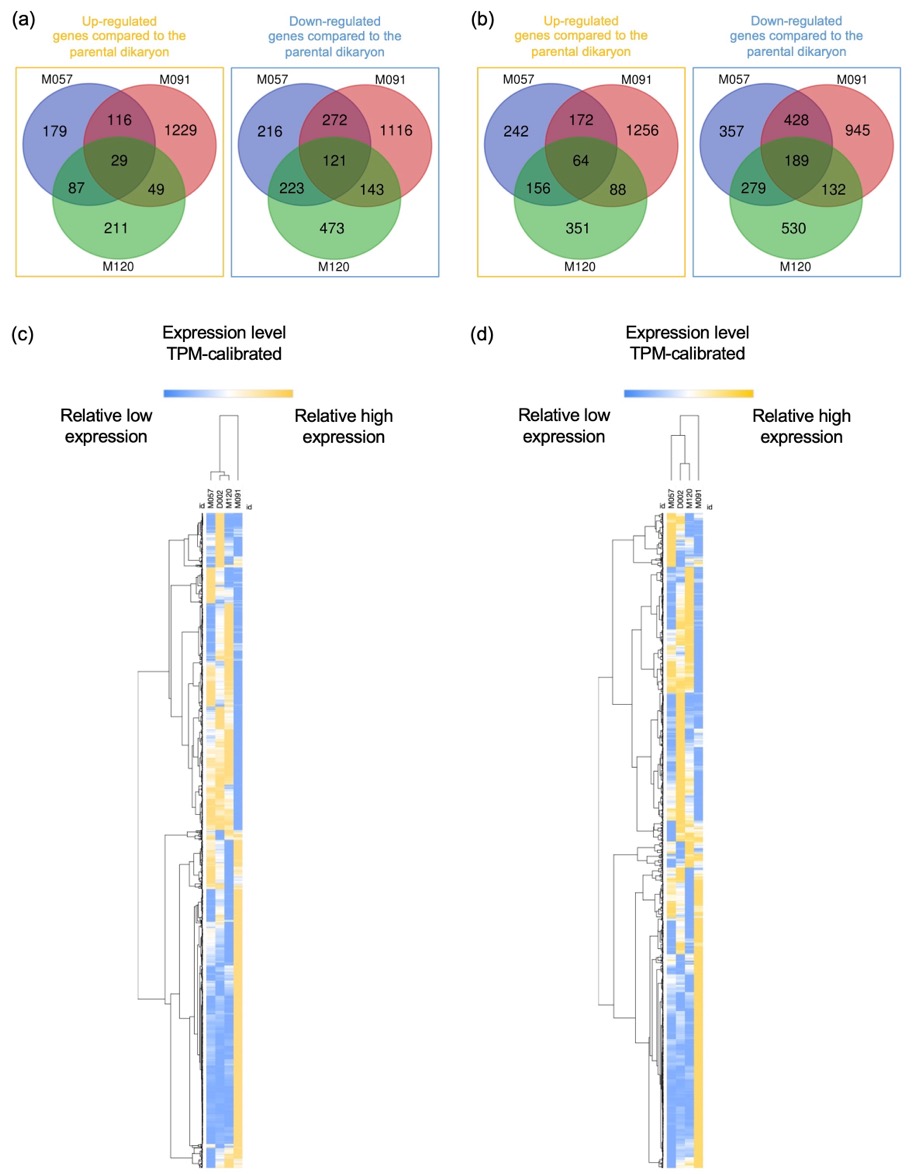


**Fig. S9 Patterns of gene regulation in *Pisolithus microcarpus* strains in different ECM and FLM tissues.** (**a–b**) Number of upregulated and downregulated genes in monokaryon strains in ECM and FLM tissues, respectively. (**c–d**) Hierarchical clustering analysis of genes significantly regulated in monokaryon strains in the ECM and FLM tissues.


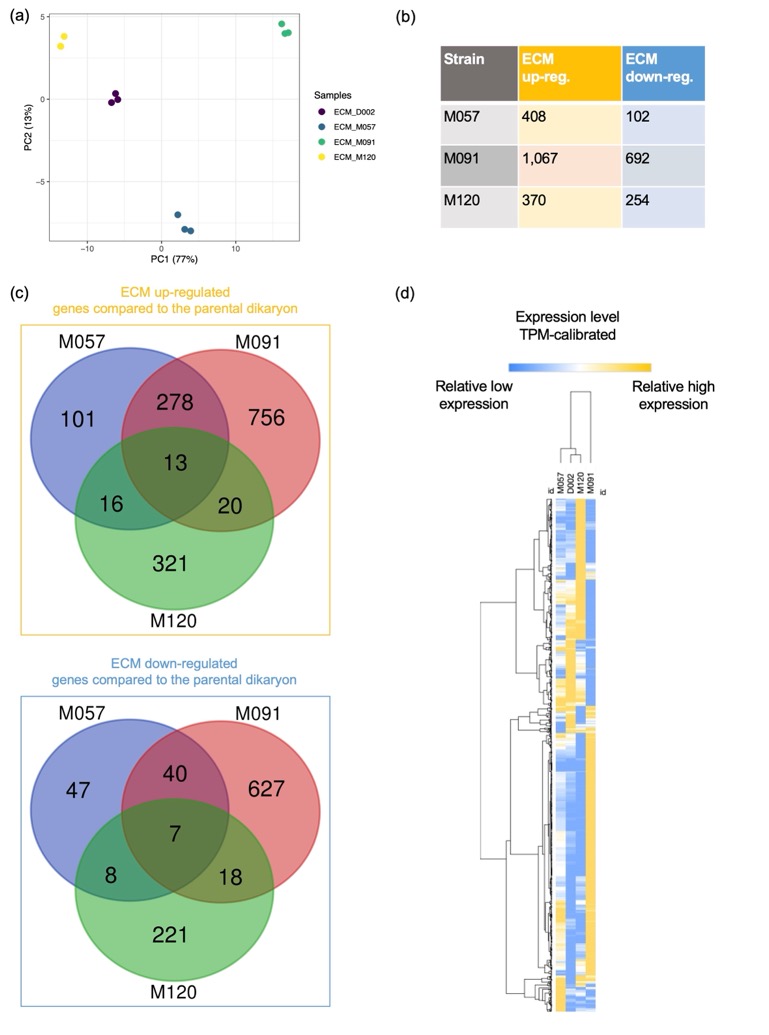


**Fig. S10 Patterns of gene regulation in *Populus* roots in contact with *Pisolithus microcarpus* strains.** (**a**) Transcriptomic profiles of ECM tissues for the poplar clone based on PCA. Colours correspond to the strains that interact with the poplar clone. (Table S2). (**b**) Number of up- and downregulated genes in poplar roots interacting with different monokaryons. (**c**) Comparison of up- and downregulated gene expression in ECM tissues or fine-root tips (M091) of poplar interacting with monokaryons relative to the parental dikaryon. Venn diagrams show the overlap of significantly regulated genes for all possible combinations. (**d**) Hierarchical clustering analysis of genes significantly regulated in ECM or fine-root tips (M091) of poplar interacting with the monokaryons.


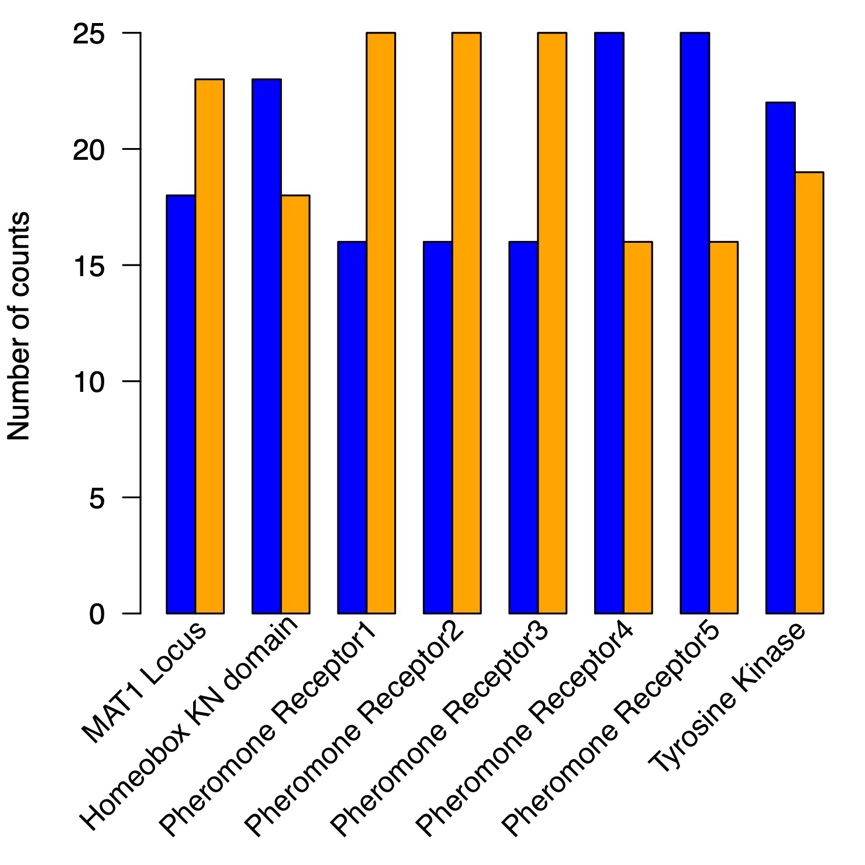


**Fig. S11** **Allele segregating at eight mating type loci in *Pisolithus microcarpus* sibling monokaryons.** Allele counts for each mating type-related gene. Blue refers to allele “A” and orange colour to allele “B”, as defined from the reference genome.

**Table S1 Description of the ectomycorrhizal traits analysed in this study.**

| **Phenotypic trait** | **Abbreviation** | **Unit** | **Type** |
| --- | --- | --- | --- |
| Number of ectomycorrhizas on average | Nb.Myco | − | Mycorrhizas counting |
| Percentage of ectomycorrhizas on average | Perc.Myco | % | Mycorrhizas counting |
| Number of lateral root tips on average | Nb.Lateral.Roots | − | Inhibition of poplar roots |
| Length of root tips and ectomycorrhizas | Root.Tip.Length | mm | Mycorrhizas anatomy |
| Diameter of lateral root tips and ectomycorrhizas | Root.Tip.Diameter | μm | Mycorrhizas anatomy |
| Mantle thickness | Mantle.Thickness | μm | Mycorrhizas anatomy |

**Table S2 Phenotypic variation for each *Pisolithus microcarpus* strain analysed in this study.** The mean values of each strain were calculated for all replicates. The parental dikaryon and monokaryons are noted with “D” and “M”, respectively.

| **Sample.**  **ID** | **Nb.**  **Myco** | **Perc.**  **Myco** | **Nb.Lateral.**  **Roots** | **Root.Tip.**  **Length** | **Root.Tip.**  **Diameter** | **Mantle.**  **Thickness** |
| --- | --- | --- | --- | --- | --- | --- |
| D002 | 25.50 | 22.19 | 118.50 | 2.41250 | 997.50000 | 72.50000 |
| M005 | 19.10 | 20.19 | 103.45 | 2.38500 | 92.50000 | 40.00000 |
| M016 | 13.40 | 18.07 | 69.35 | 2.08750 | 477.50000 | 40.00000 |
| M034 | 14.35 | 11.01 | 130.40 | 2.91500 | 687.50000 | 72.50000 |
| M039 | 0.00 | 0.00 | 81.33 | 0.60000 | 35.00000 | 0.00000 |
| M041 | 0.90 | 1.04 | 81.15 | 0.81250 | 65.00000 | 0.00000 |
| M042 | 0.30 | 0.38 | 45.55 | 5.13000 | 320.00000 | 0.00000 |
| M045 | 10.60 | 13.97 | 82.95 | 0.46000 | 175.00000 | 30.00000 |
| M050 | 7.00 | 33.93 | 21.33 | 1.28000 | 80.00000 | 87.50000 |
| M051 | 0.56 | 4.89 | 7.78 | 2.40750 | 527.50000 | 35.00000 |
| M052 | 8.50 | 12.43 | 65.78 | 2.13500 | 355.00000 | 75.00000 |
| M057 | 32.33 | 28.54 | 117.61 | 1.52750 | 387.50000 | 55.00000 |
| M058 | 8.72 | 27.89 | 31.00 | 0.84000 | 230.00000 | 67.50000 |
| M059 | 10.78 | 17.53 | 64.83 | 0.69250 | 290.00000 | 107.50000 |
| M060 | 0.25 | 3.07 | 8.95 | 3.13250 | 195.00000 | 95.00000 |
| M061 | 0.55 | 0.31 | 120.55 | 4.56500 | 270.00000 | 95.00000 |
| M063 | 0.00 | 0.00 | 86.38 | 2.46750 | 107.50000 | 42.50000 |
| M063A | 25.28 | 33.74 | 73.44 | 0.81500 | 135.00000 | 6.33225 |
| M066 | 10.63 | 13.15 | 74.05 | 2.33000 | 82.50000 | 100.00000 |
| M067 | 5.67 | 9.03 | 30.00 | 1.31250 | 80.00000 | 87.50000 |
| M068 | 1.20 | 9.55 | 6.70 | 1.63500 | 55.00000 | 0.00000 |
| M070 | 9.75 | 14.47 | 71.69 | 2.23000 | 722.50000 | 0.00000 |
| M075 | 2.50 | 3.96 | 51.85 | 0.55000 | 280.00000 | 0.00000 |
| M079 | 0.00 | 0.00 | 85.29 | 1.47250 | 135.00000 | 40.00000 |
| M080 | 11.72 | 19.75 | 55.28 | 1.99250 | 132.50000 | 17.50000 |
| M081 | 8.20 | 15.57 | 53.05 | 1.52000 | 42.50000 | 30.00000 |
| M082 | 0.00 | 0.00 | 85.43 | 1.11250 | 142.50000 | 0.00000 |
| M083 | 13.28 | 27.63 | 45.56 | 4.88250 | 687.50000 | 37.50000 |
| M091 | 0.00 | 0.00 | 131.57 | 1.91375 | 120.00000 | 0.00000 |
| M095 | 5.44 | 10.05 | 88.75 | 2.55000 | 687.50000 | 17.50000 |
| M096 | 7.75 | 9.33 | 82.75 | 4.27000 | 230.00000 | 25.00000 |
| M097 | 6.56 | 13.02 | 52.06 | 2.42000 | 27.50000 | 20.00000 |
| M098 | 1.39 | 2.87 | 21.78 | 2.94750 | 307.50000 | 20.00000 |
| M103 | 22.56 | 29.98 | 76.19 | 1.07000 | 175.00000 | 67.50000 |
| M106 | 18.90 | 28.21 | 66.90 | 0.73000 | 925.00000 | 47.50000 |
| M111 | 14.13 | 47.18 | 33.00 | 2.96500 | 140.00000 | 92.50000 |
| M117 | 33.15 | 37.22 | 87.75 | 0.96000 | 560.00000 | 45.00000 |
| M118 | 6.40 | 8.05 | 67.25 | 2.24500 | 122.50000 | 0.00000 |
| M119 | 9.00 | 13.14 | 70.70 | 1.87750 | 45.00000 | 30.00000 |
| M120 | 36.70 | 67.95 | 55.90 | 2.49250 | 372.50000 | 72.50000 |
| M127 | 1.56 | 10.91 | 10.94 | 1.97000 | 340.00000 | 30.00000 |

**Table S3 Summary of genome sequencing data with mapping statistics from the studied *Pisolithus microcarpus* strains.**

| **Strain** | **Number of raw reads** | **Number of reads after trimming** | **Number of mapped reads** | **Percent of mapped reads** |
| --- | --- | --- | --- | --- |
| D002 | 9'011'500 | 8'635'962 | 7'456'536 | 86.34 |
| M005 | 31'303'458 | 30'040'908 | 26'832'763 | 89.32 |
| M016 | 11'944'800 | 11'533'308 | 10'403'974 | 90.21 |
| M034 | 27'014'650 | 22'708'954 | 18'898'516 | 83.22 |
| M039 | 28'922'404 | 27'741'848 | 24'241'542 | 87.38 |
| M041 | 22'609'074 | 20'175'636 | 17'106'646 | 84.79 |
| M042 | 20'915'418 | 19'008'248 | 16'112'725 | 84.77 |
| M045 | 14'432'802 | 13'822'334 | 12'166'564 | 88.02 |
| M050 | 21'984'772 | 20'835'310 | 18'227'832 | 87.49 |
| M051 | 43'706'856 | 42'260'738 | 36'751'812 | 86.96 |
| M052 | 22'665'482 | 21'303'084 | 17'301'467 | 81.22 |
| M057 | 20'934'758 | 19'617'670 | 17'049'446 | 86.91 |
| M058 | 24'987'422 | 22'791'410 | 20'086'400 | 88.13 |
| M059 | 22'575'902 | 21'723'960 | 19'360'044 | 89.12 |
| M060 | 23'135'588 | 22'032'396 | 18'765'456 | 85.17 |
| M061 | 19'590'364 | 18'673'290 | 16'819'494 | 90.07 |
| M063 | 35'645'410 | 31'352'534 | 28'132'892 | 89.73 |
| M063A | 15'976'132 | 14'290'940 | 12'592'372 | 88.11 |
| M066 | 24'883'116 | 23'812'590 | 21'149'217 | 88.82 |
| M067 | 27'648'872 | 26'454'126 | 22'982'443 | 86.88 |
| M068 | 25'871'980 | 24'611'512 | 21'303'635 | 86.56 |
| M070 | 24'384'340 | 23'126'542 | 19'093'305 | 82.56 |
| M075 | 17'329'638 | 16'605'554 | 14'220'079 | 85.63 |
| M079 | 13'925'472 | 12'928'974 | 11'398'113 | 88.16 |
| M080 | 24'980'210 | 24'463'998 | 22'569'512 | 92.26 |
| M081 | 19'958'326 | 19'391'898 | 16'875'652 | 87.02 |
| M082 | 13'665'314 | 12'696'616 | 11'111'718 | 87.52 |
| M083 | 22'487'836 | 21'901'752 | 19'187'276 | 87.61 |
| M091 | 14'459'824 | 13'995'174 | 12'609'393 | 90.10 |
| M095 | 19'525'194 | 19'019'460 | 17'439'499 | 91.69 |
| M096 | 13'666'424 | 13'339'460 | 11'668'892 | 87.48 |
| M097 | 13'594'380 | 13'157'668 | 11'921'585 | 90.61 |
| M098 | 33'968'352 | 32'889'576 | 29'648'410 | 90.15 |
| M103 | 15'981'736 | 10'892'462 | 9'384'416 | 86.16 |
| M106 | 21'895'604 | 21'149'594 | 18'357'924 | 86.80 |
| M111 | 19'324'786 | 18'338'132 | 15'730'251 | 85.78 |
| M117 | 10'838'754 | 10'235'282 | 9'181'547 | 89.70 |
| M118 | 16'091'112 | 15'467'148 | 13'958'339 | 90.25 |
| M119 | 9'743'100 | 8'717'752 | 7'528'658 | 86.36 |
| M120 | 13'753'494 | 13'440'926 | 11'921'505 | 88.70 |
| M127 | 21'809'588 | 15'425'014 | 13'365'123 | 86.65 |
| Pismic441v1 | 222'998'826 | 213'010'267 | 204'421'806 | 95.97 |

**Table S4 Characteristics of *Pisolithus microcarpus* de novo genome assemblies analysed in this study.**

| **Strain** | **Number of contigs** | **Largest contig (bp)** | **Total length (bp)** | **GC content (%)** | **N50** | **L50** |
| --- | --- | --- | --- | --- | --- | --- |
| M005 | 2'806 | 915,642 | 37'062'831 | 49.50 | 68,107 | 108 |
| M016 | 3'344 | 916'613 | 35'940'970 | 49.56 | 64'725 | 110 |
| M034 | 2'891 | 942'081 | 36'173'416 | 49.49 | 73'026 | 96 |
| M039 | 2'638 | 1'023'729 | 36'516'767 | 49.51 | 69'860 | 101 |
| M041 | 2'765 | 916'278 | 35'514'304 | 49.56 | 72'092 | 97 |
| M042 | 2'542 | 915'353 | 35'263'012 | 49.57 | 75'468 | 98 |
| M045 | 3'181 | 924'519 | 35'967'798 | 49.54 | 71'690 | 107 |
| M050 | 2'578 | 958'440 | 35'569'465 | 49.57 | 73'239 | 95 |
| M051 | 2'457 | 916'349 | 36'710'439 | 49.50 | 68'586 | 111 |
| M052 | 2'833 | 920'530 | 36'154'536 | 49.53 | 68'265 | 104 |
| M057 | 2'675 | 915'357 | 36'366'168 | 49.51 | 68'387 | 98 |
| M058 | 2'688 | 917'177 | 36'414'835 | 49.51 | 71'965 | 109 |
| M059 | 2'894 | 623'830 | 36'478'607 | 49.53 | 68'474 | 121 |
| M060 | 2'986 | 915'406 | 36'480'555 | 49.48 | 71'738 | 105 |
| M061 | 2'738 | 872'246 | 36'053'137 | 49.53 | 79'578 | 94 |
| M063 | 2'423 | 916'649 | 35'879'312 | 49.56 | 74'198 | 102 |
| M063A | 2'958 | 843'415 | 35'512'226 | 49.57 | 68'674 | 107 |
| M066 | 2'681 | 718'290 | 36'162'966 | 49.55 | 72'938 | 108 |
| M067 | 2'614 | 891'608 | 35'571'859 | 49.59 | 73'013 | 101 |
| M068 | 2'630 | 714'225 | 36'408'439 | 49.51 | 67'916 | 110 |
| M069 | 5'417 | 710'068 | 34'187'208 | 49.66 | 39'999 | 168 |
| M070 | 2'861 | 916'847 | 36'141'386 | 49.54 | 71'965 | 109 |
| M075 | 2'748 | 727'692 | 35'192'159 | 49.56 | 76'440 | 101 |
| M079 | 3'064 | 880'408 | 35'464'925 | 49.56 | 70'167 | 106 |
| M080 | 2'578 | 828'108 | 35'882'343 | 49.58 | 70'576 | 103 |
| M081 | 2'802 | 969'676 | 36'734'645 | 49.47 | 68'626 | 103 |
| M082 | 3'137 | 938'140 | 35'845'086 | 49.57 | 69'000 | 107 |
| M083 | 2'573 | 819'278 | 36'261'487 | 49.53 | 70'129 | 100 |
| M091 | 2'858 | 925'671 | 36'150'926 | 49.53 | 70'835 | 102 |
| M095 | 2'668 | 728'673 | 35'465'567 | 49.61 | 79'032 | 102 |
| M096 | 3'167 | 941'025 | 35'796'414 | 49.55 | 69'224 | 106 |
| M097 | 3'196 | 916'547 | 36'074'509 | 49.54 | 66'377 | 113 |
| M098 | 2'507 | 871'206 | 36'398'411 | 49.55 | 79'100 | 99 |
| M103 | 3'437 | 691'354 | 35'827'888 | 49.54 | 59'610 | 118 |
| M106 | 2'607 | 942'077 | 36'033'193 | 49.54 | 79'806 | 92 |
| M111 | 2'987 | 941'027 | 36'057'292 | 49.53 | 70'552 | 101 |
| M117 | 3'209 | 876'542 | 35'532'888 | 49.56 | 64'756 | 108 |
| M118 | 3'061 | 951'902 | 35'899'912 | 49.59 | 66'960 | 119 |
| M119 | 3'905 | 714'306 | 35'170'855 | 49.58 | 60'712 | 115 |
| M120 | 3'334 | 694'322 | 36'382'754 | 49.51 | 63'527 | 113 |
| M127 | 2'909 | 941'027 | 34'927'780 | 49.60 | 74'163 | 89 |

**Table S5 Number of significant associations between CNV regions and ECM traits from the studied *Pisolithus microcarpus* strains using generalised additive models.**

| **Trait** | **Nb. significant associations** | **Mean of *R*^2^** | **Minimum of *R*^2^** | **Maximum of *R*^2^** |
| --- | --- | --- | --- | --- |
| Nb.Myco | 0 | – | – | – |
| Perc.Myco | 15 | 0.104479851 | 0.076142403 | 0.148342086 |
| Nb.Lateral.Roots | 39 | 0.148304221 | 0.087761889 | 0.203912925 |
| Root.Tip.Length | 3 | 0.150871815 | 0.080908212 | 0.227855428 |
| Root.Tip.Diameter | 21 | 0.107723859 | 0.082142975 | 0.148544031 |
| Mantle.Thickness | 36 | 0.15667552 | 0.07561405 | 0.179275032 |

**Table S6 Complete list of upregulated genes in ECM tissues of D002, M057, and M120 compared to their respective FLM tissues.** The upregulated genes also upregulated in M091 were excluded to restrict the top-candidate to symbiosis-related genes. The complete list is embedded into the excel file GenTransSymbiosisPisolithus_TableS6.xlsx
